# Somato-dendritic morphology and dendritic signal transfer properties differentiate between fore- and hindlimb innervating motoneurons in the frog *Rana esculenta*

**DOI:** 10.1186/1471-2202-13-68

**Published:** 2012-06-18

**Authors:** András Stelescu, János Sümegi, Ildikó Wéber, András Birinyi, Ervin Wolf

**Affiliations:** 1 Department of Anatomy, Histology and Embryology, Faculty of Medicine, Medical and Health Science Center, University of Debrecen, Nagyerdei krt 98, Debrecen, H-4032, Hungary

## Abstract

**Background:**

The location specific motor pattern generation properties of the spinal cord along its rostro-caudal axis have been demonstrated. However, it is still unclear that these differences are due to the different spinal interneuronal networks underlying locomotions or there are also segmental differences in motoneurons innervating different limbs. Frogs use their fore- and hindlimbs differently during jumping and swimming. Therefore we hypothesized that limb innervating motoneurons, located in the cervical and lumbar spinal cord, are different in their morphology and dendritic signal transfer properties. The test of this hypothesis what we report here.

**Results:**

Discriminant analysis classified segmental origin of the intracellularly labeled and three-dimensionally reconstructed motoneurons 100% correctly based on twelve morphological variables. Somata of lumbar motoneurons were rounder; the dendrites had bigger total length, more branches with higher branching orders and different spatial distributions of branch points. The ventro-medial extent of cervical dendrites was bigger than in lumbar motoneurons. Computational models of the motoneurons showed that dendritic signal transfer properties were also different in the two groups of motoneurons. Whether log attenuations were higher or lower in cervical than in lumbar motoneurons depended on the proximity of dendritic input to the soma. To investigate dendritic voltage and current transfer properties imposed by dendritic architecture rather than by neuronal size we used standardized distributions of transfer variables. We introduced a novel combination of cluster analysis and homogeneity indexes to quantify segmental segregation tendencies of motoneurons based on their dendritic transfer properties. A segregation tendency of cervical and lumbar motoneurons was detected by the rates of steady-state and transient voltage-amplitude transfers from dendrites to soma at all levels of synaptic background activities, modeled by varying the specific dendritic membrane resistance. On the other hand no segregation was observed by the steady-state current transfer except under high background activity.

**Conclusions:**

We found size-dependent and size-independent differences in morphology and electrical structure of the limb moving motoneurons based on their spinal segmental location in frogs. Location specificity of locomotor networks is therefore partly due to segmental differences in motoneurons driving fore-, and hindlimbs.

## Background

Investigation and comparison of morphological and electrical properties of *different* neurons and the search for their functional implications have been a challenge in neurobiology since the very early stages. Correlative analysis of the *same type* of neuron *in different parts* of the CNS has shown location specific morphological and electrotonic differences in pyramidal neurons of the hippocampus in the rat [1]. In our current paper we report location specific properties of a subclass of another CNS neuron, the limb moving alpha motoneurons (MNs) in the cervical and lumbar spinal cord of frogs (*Rana esculenta*) supplying the muscles of forelimbs and hindlimbs. These MNs are especially suitable for this kind of analysis since they provide different patterns of movements for the forelimbs and hindlimbs of semi-aquatic frogs in terrestrial and in water locomotion. In general, frogs jump and swim rather than walk. During the upward movement of jumping through the air the forelimbs are retracted and adducted close to the side of the body, but as the animal begins to fall, the forelimbs are protracted in readiness to break the impact of the body on reaching the ground. During swimming frogs employ the hindlimbs as paddles with a major role in propulsive impulse production and forelimbs are used only as thrusters in directional changes. The movements of the hindlimbs during swimming are very much like those performed in jumping; they are drawn up in the form of a Z and quickly extended producing an intensive stroke. This way, the hindlimbs innervated by lumbar MNs play the chief role in locomotion without major differences between the kinematics of jumping and swimming in *Rana esculenta*[2]. The ilio-sacral group of muscles is active the most during the take-off phase of jump and during the propulsive phase of swimming and the firing pattern of this muscle group was found to be similar during swimming and jumping [3,4]. These similarities in kinematics of movements of the hindlimbs and in firing patterns of major hindlimb-moving muscles that are active during different forms of locomotions nicely illustrate that major physiological properties of hindlimb moving MNs well suit the activity of musculature during both types of locomotions. However, frogs use their fore- and hindlimbs rather differently. Therefore, taking the thoughts of MN adaptation seriously, one may end up with the hypothesis that the major physiological properties of limb moving MNs may tend to be different for the forelimbs and hindlimbs. Since MNs destined to move muscles in fore- and hindlimbs of frogs are segregated in the cervical and lumbar spinal cord respectively, and morphology and electrotonic properties of these MNs are expected to have high impact on their physiological properties, we expected morphological and electrical differences between limb moving MNs in a location dependent manner along the spinal cord. The test of this proposal is what we report in this paper.

The location specific motor pattern generation properties of the spinal cord along its rostro-caudal axis were clearly demonstrated in experiments with newt embryos. In these experiments, when grafts of limb innervating cervical and lumbo-sacral spinal cord segments were replaced by each other, the rhythm, coordination, and general characters of limb movements were determined by the innervating spinal segments irrespective of their heterotopic nature to the innervated limb [5]. Specificity of the brachial spinal cord that innervates wings in chicks was also demonstrated by similar experiments [6,7]. However, it was impossible to draw any conclusion from these experiments as to the possible morphological and electrical differences in MNs located in different spinal segments that innervate different limbs. Our current study was focused on the investigation of such location-dependent (segmental) differences in geometry, orientation and passive electrical properties of limb moving MNs in the cervical and lumbar spinal cord.

## Methods

### The sample of motoneurons

In this study a sample of eight cervical and eight lumbar limb innervating alpha MNs of adult frogs (*Rana esculenta*) was used. The MNs were intracellularly labeled in previous experiments and three-dimensionally reconstructed in our laboratory from serial sections of the spinal cord by using Neurolucida ver. 2 (Microbrightfield, USA) or its predecessor, a computer aided microscope system. The cervical MNs were located in the third segment and the lumbar MNs were situated in the eighth or in the rostral part of the ninth segment of the spinal cord. The limb innervating type of these MNs was identified on the basis of the shape and lateral motor column location of their cell bodies and the characteristic arborization pattern and extension of the dendrites [8]. Within the lateral motor column, more medial MNs innervate ventral, while lateral MNs the dorsal limb muscles [9]. Cell bodies of MNs used in the recent study were all located in the lateral motor column but in different medio-lateral positions without any bias. Therefore, our sample must have contained MNs innervating different muscles of the fore- or hindlimbs. This way we pooled limb moving MNs to investigate their features specific to the cervical and lumbar segments where they were located rather than to the muscles they innervated. For such segment specific differences between the MNs we will use the term ‘segmental differences’ throughout the paper.

Details about the labeling procedures, tissue preparations, shrinkage and optical corrections of morphological data were described in the original papers [10,11].

### Morphometry

To characterize the MNs quantitatively, twelve morphological variables were used. These morphological variables may be divided into three groups describing the soma, the stem dendrites and the rest of the dendritic trees (Table 1).

**Table 1:** Metric morphological data measured and their description

**Group**	**Morphological variable**	**Description**
**Soma**	Roundness	The ratio between the maximum and minimum diameters of the soma.
	Soma surface	The average surface area of the prolate and oblate ellipsoids fitted to the soma.
**Stem dendrites**	Number of stem dendrites	Number of dendrites connected to the soma.
	Sum of diameters of stem dendrites	The mean stem diameter multiplied by the number of stem dendrites.
**Dendritic tree**	Number of dendritic branches	A branch is defined as part of the dendritic tree between two branch points, a branch point and an end point or between the soma and the first branch point.
	Maximum order of dendritic branches	The highest number of branch points along dendritic paths from the soma to end points.
	Combined (total) dendritic length	Sum of the length of all dendritic branches.
	Surface	Sum of the surface of all dendritic branches.
	Mean parent length	Mean length of branches connecting two branch points in the dendritic tree of the neuron.
	Mean distance to BRP	Mean distance of branch points from the soma measured along the dendritic branches (path distance).
	Mean distance to ENDP	Mean distance of end points from the soma measured along the dendritic branches (path distance).
	Max distance of ENDP	Path distance of the farthest end point from the soma.

Descriptors were divided into three groups describing the somata, stem dendrites and dendritic arborization.

Perikarya of MNs innervating limb muscles have ellipsoid or fusiform shapes both in the lumbar and cervical spinal cord. Calculation of surface area of somata was based on the major and minor diameters of somata measured on photographs of the perikarya at 500X magnification, which were corrected for tissue shrinkage [10]. Then the average surface area of the prolate and oblate ellipsoids fitted to somata was calculated and regarded as an estimate for the surface area of the cell body [12]. Quantitative morphological parameters for dendrites were obtained directly from the data files containing the reconstructed geometry of dendrites.

### Computer modeling and descriptors of signal transfer properties

#### Discretization of the model

We used the simulation environment of NEURON [13,14] to create high-fidelity compartmental cable models of the MNs based on the reconstructed geometry of dendrites and somata. We simulated the dendritic impulse propagation, measured attenuations of signals during their propagation along the dendrites to the soma and visualized the morphoelectrotonic transforms (MET) of dendrites (see later) by using our own hoc codes to command NEURON. The simulations were run under MS Windows Professional on Pentium IV PCs and integration time step of 0.025 ms was used. A new cylindrical compartment was always started in the cable model when the dendrites branched, changed their diameter or ended. To increase computational accuracy, a maximum possible length of compartments was set and if a compartment of the model was longer than this value, the compartment was subdivided into more sub-compartments. Our choice for the longest possible compartment was based on the criterion established by comparing results of analytical calculations and compartmental models of the same dendritic trees. If the length of the longest compartment did not exceed 20% of the space constant (λ), then the error imposed by the compartmentalization of dendrites was physiologically irrelevant [15]. Since the space constant is proportional to the square root of the specific dendritic membrane resistance and the square root of the diameter, we used the hardest criterion calculated for the smallest calibre (0.3 μm) dendrites with the lowest dendritic resistance we used (5000 Ω·cm^2^). In this case the 0.2 λ criterion yielded 38 μm as the maximum geometrical length of compartments, what we used in all simulations. The number of compartments ranged between 880 and 6209 per neuron depending on neuronal size and complexity.

#### Membrane properties

We assumed passive membrane with biophysical properties measured for similar MNs. The mean input resistance at the soma and the axial resistivity of the cytoplasm of spinal MNs in frogs were measured to be 1.4 ± 0.7 MΩ [16] and 110 Ω·cm respectively (see [17], p 44), and these are the values that we adopted. There are also much higher input resistances measured by intracellular electrodes ranging from 1.9 to 6.0 MΩ [18,19]. To account for these higher values, in some simulations we investigated MNs with 5 MΩ input resistance. A wealth of data suggests that the distribution of specific membrane resistance is inhomogeneous over the soma-dendrite surface and it is bigger for the dendrites than for the soma in many types of neurons including MNs [20-28]. However, the estimates for the specific membrane resistances and for the degree of inhomogeneity between the soma and dendrites vary in a wide range [29-31]. In addition, the effective membrane resistance is constantly changing due to the ever changing activation state of the tens of thousands of synapses received by these cells [10]. To account for these features, we have conducted simulations by assuming both homogeneous and inhomogeneous soma-dendrite membranes and checked our results against variations in membrane properties. In the case of homogeneous membrane the specific membrane resistance was the same for the soma and the dendrites (R_ms_ = R_md_) and this common value was determined by the compartmental model so that the input resistance at the soma had 1.4 MΩ or 5 MΩ values. In the inhomogeneous model we assumed a step increase in membrane resistance towards the dendrites at the soma-dendritic junction (R_ms_ < R_md_) but the membrane was uniform over the dendrites and the soma [29,30]. This step increase in membrane resistance is a simplification as opposed to a continuous change in membrane resistance of dendrites with the distance from the soma. However, Fleshman et al. [25] and Segev et al. [32] could not find any physiologically relevant difference between the step model and a model with continuously changing dendritic membrane resistance based on fitting morphologically realistic models to physiological data. In our inhomogeneous models (R_ms_ < R_md_) the specific dendritic membrane resistance was fixed and the resistance of the soma was determined by using the computer model to reproduce the physiologically realistic input resistance of the MN. In simulations when the effects of changes in the general level of synaptic activity received by dendrites (background synaptic activity of dendrites) were investigated, the specific membrane resistance of dendrites was varied and the somatic specific membrane resistance was kept constant [29,33,34]. In these simulations we used 5000, 20000 and 50000 Ω·cm^2^ specific resistances for dendrites to mimic high, middle (control) and low levels of background synaptic activities and the somatic resitance was 500 Ω·cm^2^. With these R_md_-R_ms_ pairs the somatic input resitance remained within its physiological range [16,18,19] for all MNs. Resting potentials of MNs were set to −75 mV, the reversal potential of the synapses was 0 mV and the specific membrane capacitance was 1 μF·cm^2^.

#### Initiation of PSPs and measures of dendritic signal transfer

To analyze MNs electrotonically, different measures of signal transfers were computed between dendritic points and the soma in the various membrane models of MNs by using the NEURON software [13,14]. Steady-state voltage- and current transfers and the log attenuation of voltage were investigated while a constant current was injected to midpoints of dendritic compartments and voltage or current recorded at the midpoint of the soma compartment. Steady-state voltage (current) transfers were defined by the somatic voltage (current reaching the soma) divided by the voltage (current injected) at the dendritic site. Log attenuation of voltage was defined as the logarithm of the ratio of voltage-time integrals at the dendrite and at the soma [35]. Propagation of transient signals was studied by measuring transfers of voltage generated by conductance changes according to an alpha function [17] with 2 nS amplitude and with 1.5 ms rise time to its maximum to model local synaptic activity in the midpoints of dendritic compartments. This kinetics of the conductance change mimics single fiber EPSPs measured in monosynaptic reticulospinal axon to MN connections [30,36]. During the transfer of voltage transients towards the soma the amplitude, half-width and 10–90% rise time of PSPs were measured. The propagation of voltage transients were described by the ratio of the somatic and dendritic values of these shape parameters (somatic amplitude/dendritic amplitude, somatic half-width/dendritic half-width and somatic rise time/dendritic rise time).

**Figure 1:**
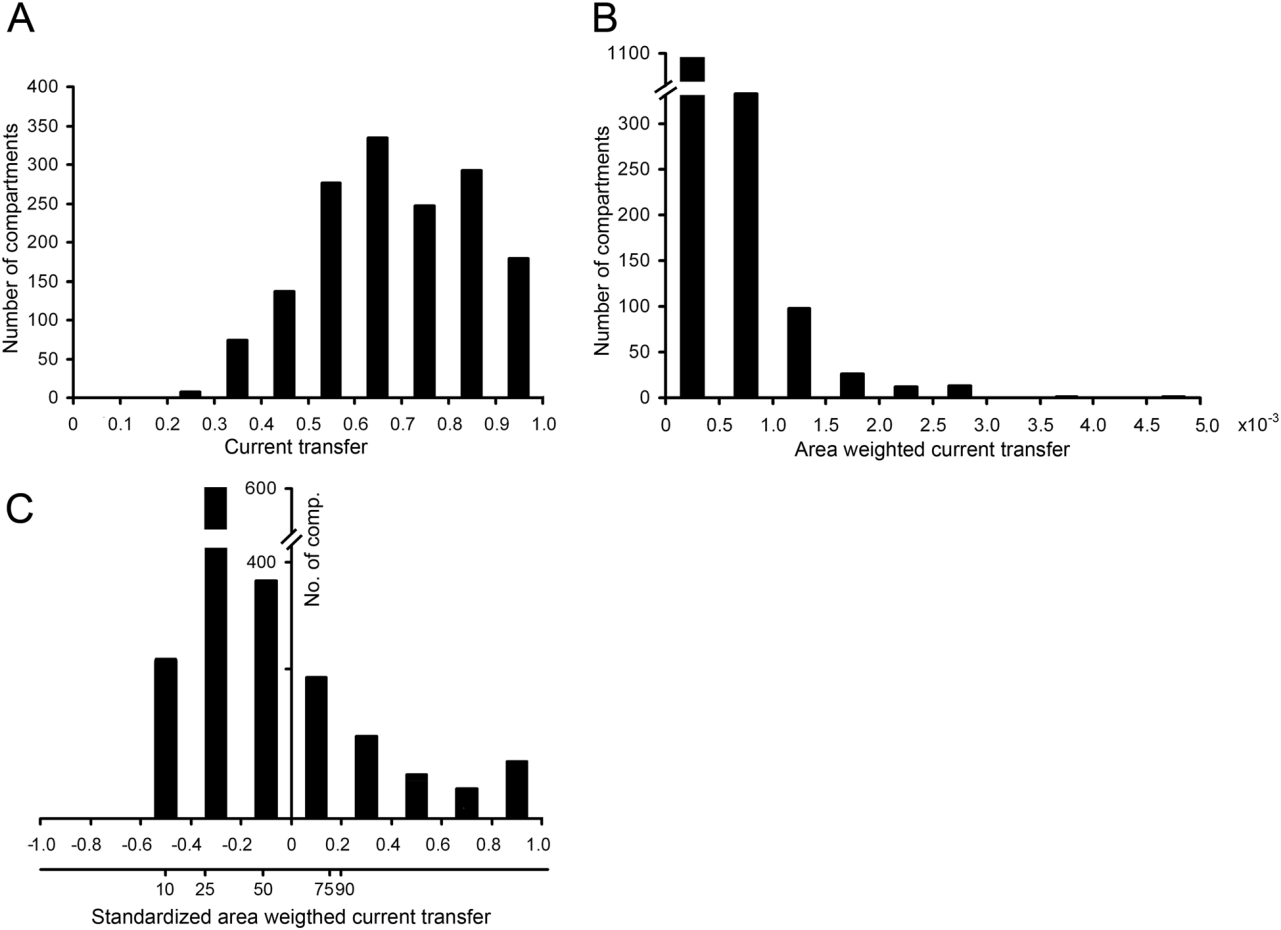
**Steps of data processing to create standardized area weighted distributions of signal transfer values.** Example is based on current transfers of MN C-IC82 but steps are similar for other measures of signal transfers we used. (**A**) Frequency distribution of somatopetal current transfers measured from mid-points of each compartment. These raw values were area weighted (**B**) to give proportionally bigger weight to transfers from compartments with bigger surface area. Then the area weighted distribution was standardized (**C**) to eliminate variable size effects of MNs on signal transfers. Shape of standardized and area weighted distribution of transfers was quantified by the 10^th^, 25^th^, 50^th^, 75^th^ and 90^th^ percentiles of the distribution (see lower horizontal axis in part **C**).

In our investigations current and voltage transfers as well as somatic to dendritic ratios of shape parameters of transient EPSPs were weighted by the surface of the dendritic compartment whose mid-point was used to generate the signal (see Figure 1A–B for illustration). Area weighting is useful to give proportionally higher weight for transfer values that approximate transfers of PSPs of more synapses received by a bigger dendritic compartment. This area weighting is especially appropriate for spinal MNs of frogs where it was shown that areal synaptic density (the number of synapses received by a unit dendritic surface) is the same over the whole dendritic arborization, independently of the distance from the soma and the diameter of the dendrite [37]. This way the area of a compartment is directly proportional to the number of synapses received by that compartment. Area weighted voltage and current transfers were then standardized (Figure 1C). Standardization is a well-known mathematical transformation that replaces each area weighted measurement by its sample standard score (z score) so that distributions have a mean value of zero and a standard deviation of 1. E.g. if X is an area weighted transfer value then its z score becomes (X-μ)/σ, where μ and σ are the mean and standard deviation of the distribution of X values. So z scores indicate how far above or below the mean a given score in the distribution is in standard deviation units. Standardization preserves the shape of area weighted distributions while differences due to variance in size of MNs are eliminated. This allowed comparison of shapes of signal transfer distributions independently of the size differences of MNs. Shape of these standardized distributions were described by their 10^th^, 25^th^, 50^th^, 75^th^ and 90^th^ percentiles; the values in the distributions, below which the corresponding percents of all observations fall. Since the frequency of dendrites with very low and high transfer values to soma is limited, the errors associated with increasingly lower and higher percentiles are disproportionally bigger than the error associated with the 50^th^ percentile [34]. In order to compensate for these percentile dependent errors, percentiles were weighted relative to the 50^th^ percentile (or median value, whose weight was 1). Weighting process was performed by two different sets of weighting factors to check if our results are independent on the particular weighting strategy. First, the 10^th^, 90^th^ and 25^th^, 75^th^ percentiles had 0.2 and 0.8 weights respectively, while in the second case, the weights were 0.33 and 0.67. The two sets of these weighted percentiles were then used as descriptors in hierarchical cluster analysis to classify MNs based on their dendritic signal transfer properties and the percentiles are shown as box plots in figures. 

### Statistical analysis

For statistical analysis and plotting the figures the Microsoft Office (Microsoft Corp.), PAST [38] and SPSS (SPSS Inc., Chicago IL) softwares were used. In paired comparisons of means either the two-tailed *t*-test or the Mann–Whitney test was used depending on whether the criteria for using a *t*-test were met. Normality of distributions and equivalence of variances were tested by the Shapiro-Wilk and F-tests. Distributions of branch points were compared by the Wilcoxon signed rank test. Significance level was chosen to be 0.05 in all statistical tests. In quantitative data means are followed by standard errors of means (S.E.M.) that were also used as error bars in the figures. For non pair-wise comparisons the multivariate statistics; hierarchical cluster analysis [39] or discriminant analysis [40] was chosen. Discriminant analysis was used in metric analysis of dendritic arbors since this method allowed identification of those properties which differentiate MNs in the cervical and lumbar spinal cord significantly. The metric descriptors used in these investigations are listed in Table 1 and Table 2. To validate the results obtained by the discriminant analysis we used different techniques. First, we reduced the number of descriptors to see if MNs are classified correctly with a reduced number of descriptors. Second, two kinds of cross-validation techniques were applied; the leave-one-out (16-fold cross validation) and the repeated random sub-sampling (ten times) with eleven MNs as training data set and the rest of five neurons as validation data set [40]. 

**Table 2:** Quantitative parameters of morphoelectrotonic transforms (METs)

	**MET descriptor**	**Cervical MNs**		**Lumbar MNs**	
		**R**_**N**_ **= 1.4 MΩ**	**R**_**N**_ **= 5 MΩ**	**R**_**N**_ **= 1.4 MΩ**	**R**_**N**_ **= 5 MΩ**
**Homogeneous membrane**	Combined MET length (λ)	439.9 ± 66.2 *	260.7 ± 25.6*	819.8 ± 130.4 *	434.7 ± 32.5 *
	Mean length (λ)	3.6 ± 0.6	2.1 ± 0.2	3.7 ± 0.5	2.0 ± 0.1
	Mean parent length (λ)	2.2 ± 0.3	1.1 ± 0.1	2.2 ± 0.3	1.3 ± 0.1
	Mean distance to BRP (λ)	5.7 ± 0.8	3.4 ± 0.2	7.1 ± 1.1	4.3 ± 0.4
	Mean distance to ENDP (λ)	12.9 ± 1.9	7.3 ± 0.5	14.2 ± 2.0	8.1 ± 0.6
	Max. distance of ENDP (λ)	24.8 ± 3.7	12.9 ± 1.2	28.7 ± 4.1	14.5 ± 1.3
**Inhomogeneous membrane**	Combined MET length (λ)	181.8 ± 26.8 *	169.4 ± 16.6 *	302.5 ± 18.7*	288.0 ± 17.9 *
	Mean length (λ)	1.4 ± 0.2	1.3 ± 0.1	1.4 ± 0.1	1.3 ± 0.1
	Mean parent length (λ)	1.2 ± 0.1	0.9 ± 0.1	1.1 ± 0.1	1.1 ± 0.2
	Mean distance to BRP (λ)	4.1 ± 0.5	2.9 ± 0.1 *	4.8 ± 0.3	3.6 ± 0.2 *
	Mean distance to ENDP (λ)	6.7 ± 0.8	5.4 ± 0.2	7.4 ± 0.4	6.0 ± 0.3
	Max. distance of ENDP (λ)	9.3 ± 1.2	7.8 ± 0.4	10.5 ± 0.7	9.2 ± 0.6

MET descriptors were measured in cervical and lumbar limb moving MNs with homogeneous (R_ms_ = R_md_) and inhomogeneous (R_ms_ < R_md_) soma-dendritic membranes and with different neuron resistances. Measures of length of dendrites in METs are in units of generalized space constant (λ). Variables with statistically significant differences in pair-wise comparisons of METs of MNs from different spinal segments were marked by asterisks (Mann–Whitney test, p < 0.05). BRP and ENDP abbreviate branch points and end points, R_N_ is the neuron resistance.

In analysis of dendritic orientation, the full circle around the soma in the transverse plane of the spinal cord was divided into equal bins of 40 degree angles and the total lengths of projected dendritic arbors of the two groups of MNs within each bin was compared by Mann–Whitney tests.

Cluster analysis was applied when MNs were characterized by percentiles of standardized and area weighted voltage-, and current transfer distributions to describe dendritic signal propagation. These descriptors have no easy direct interpretation, and therefore identification of those descriptors that discriminate cervical and lumbar MNs significantly by discriminant analysis gives no further information. However, by using these standardized descriptors we could avoid the confounding effects of size-related variability among neurons and could focus on structural rather than size-dependent electrotonic properties of dendrites.

**Figure 2:**
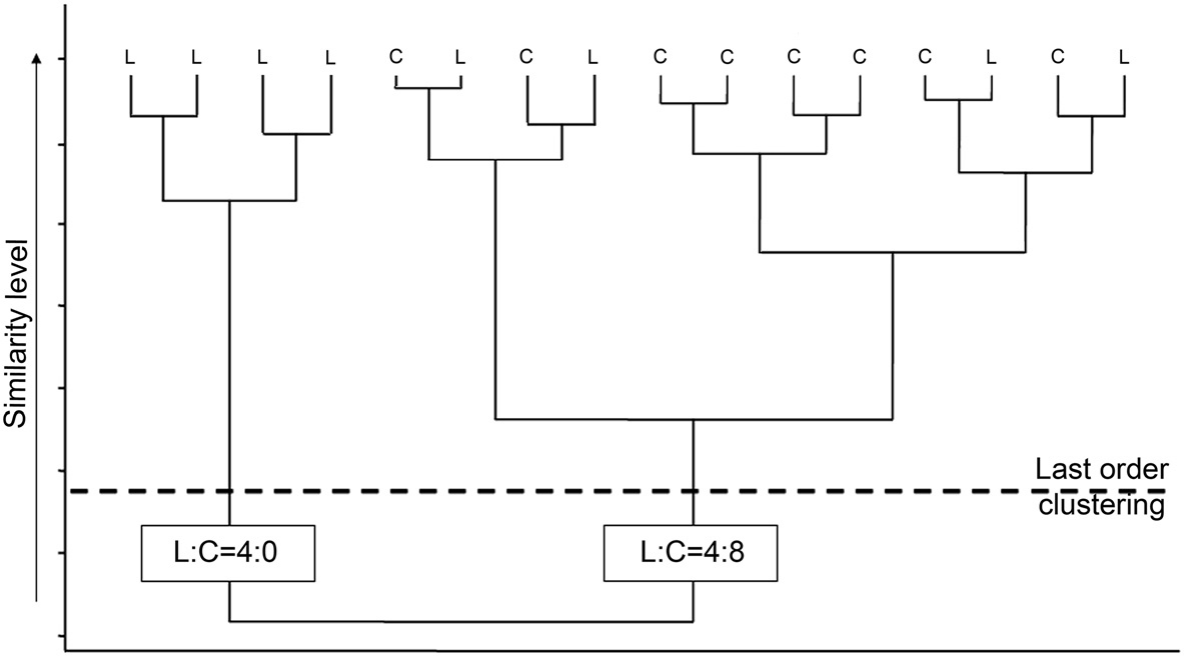
**Sample cluster formations represented by dendrogram and similarity level (dashed line) at last order clusters.** In calculation of homogeneity indexes measuring segmental homogeneities of MNs in the biggest two clusters (last order clustering and Peterson’s indexes), the ratios of MNs from the lumbar (L) and cervical (C) segments were considered in each cluster. See Methods for more details.

The cluster analysis was used with Euclidean distance metric and with two different agglomerative algorithms, the Ward’s and the Pair group methods. In the beginning of the agglomerative analysis all MNs were separated and later they were united step by step to form clusters with increasing numbers of MNs. In each consecutive agglomerative step, when further MNs or clusters of MNs were fused, the fusion occurs at increasing distances (at decreasing similarity levels). The hierarchy of agglomerative steps may be represented by a tree-like structure called dendrogram (see Figure 2 for a sample), where the smallest branches correspond to the individual MNs. During such agglomerative cluster formations, the last meaningful step is the situation when all MNs belong to two clusters just before joining all of them to a single cluster. This step with two clusters comprising all MNs what we will call last order clustering throughout this paper. Cluster formations were analyzed at this level.

To check if MNs have a tendency to form last order clusters where cervical and lumbar MNs are segregated, homogeneity (or similarity) indexes were used to measure segmental homogeneity within clusters. Measuring segmental homogeneities is feasible since increasing segmental differences among MNs are reflected in their increasing segregation tendency to different clusters shown by the cluster analysis, and as a consequence of segregation, the clusters become more and more homogeneous in terms of segmental origins of MNs they contain. This way, increasing segmental segregation of MNs between clusters may be measured by segmental homogeneities (homogeneity indexes) within clusters. Two different homogeneity indexes were used. In addition to the Peterson’s index [41] we defined another last order clustering index to investigate last order clusters. Last order clustering index was defined as the weighted average of segmental ratios (≤1) of MNs in the two last order clusters, where weighting factors were the number of neurons in clusters divided by the total number of neurons studied. E.g. if cluster A contains lumbar (L) and cervical (C) MNs in a ratio of L:C = 2:5, while in cluster B the ratio is L:C = 6:3 then, the last order clustering index becomes [(7 _*_ (2/5) + 9 _*_ (3/6)]/16 = 0.46 being the total number of neurons is 16. Another index used to measure last order cluster formations was the Peterson’s index [41] and was defined as 1 – 0.5 Σ_i_ | a_i_ – b_i_ | where a_i_ and b_i_ are the segmental portions of MNs in cluster A and B; i = lumbar or cervical. With the clusters of the above example the calculation yields: 1 – 0.5 * [| (2/7) – (6/9) | + | (5/7) – (3/9) |] = 0.62. Both indexes may have values between 0 and 1. The indexes are closer to 1 if cervical and lumbar MNs are more similar and they are getting smaller with increasing differences between MNs of the two spinal segments. The significance of segmental cluster formation tendencies (when cervical and lumbar MNs get segregated in different last order clusters) was tested by comparing these homogeneity indexes with those of artificially generated cluster formations, where segmental origins of MNs were assigned randomly prior to the cluster analysis. For each actual dendrogram 100 other artificial dendrograms were generated reflecting grouping tendencies of the real set of MNs with their segmental origins artificially randomized. The mean value of indexes was calculated for the 100 artificial dendrograms and the mean was then compared with the actually found index by one sample t-test. If this test showed a significant difference between the real and artificial indexes, then we concluded that MNs tended to form homogeneous groups determined by their segmental location in the spinal cord.

## Results

### Morphology of motoneurons

**Figure 3:**
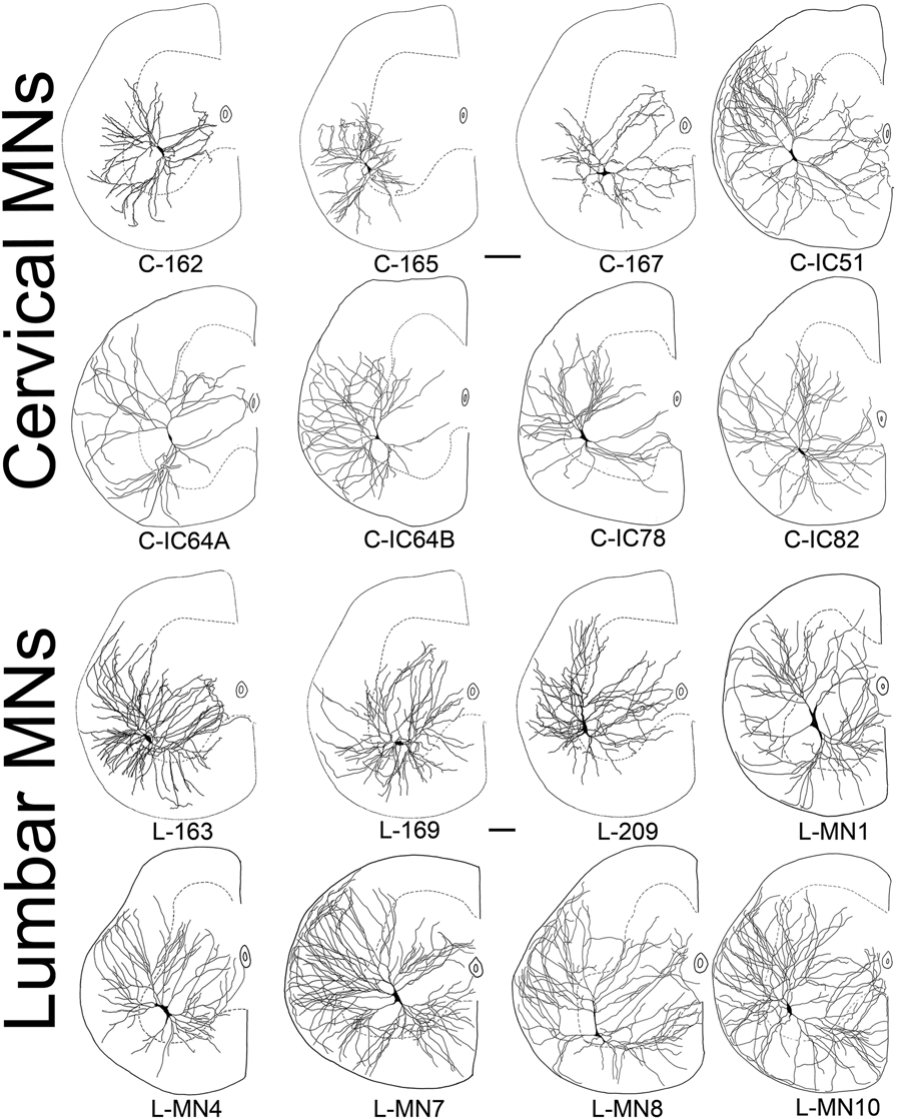
**Camera lucida drawings of dendritic trees of fore- and hindlimb moving motoneurons (MNs) of frogs.** Neurons are from the 3^rd^ cervical and the 8^th^ or 9^th^ lumbar segments as seen in the transverse plane of the spinal cord. Drawings of MNs were superimposed with the contours of spinal cord to show locations of somata and direction and extent of dendrites. Dashed lines mark the border of white and gray matters. Scale bars are 100 μm. Part of this Figure was reprinted from [10] and [11] with permissions of the publishers Elsevier and John Wiley & Sons.

The purpose of this study was to investigate differences between alpha motoneurons (MNs) located in the cervical and lumbar enlargements of the frog that innervate the muscles of forelimbs and hindlimbs. These MNs had ellipsoid or fusiform perikarya in the lateral area of the ventral horn. The dendritic arborization of these MNs could be divided into a dorsomedial, dorsal and lateral dendritic arrays with many dendrites extending to the lateral funiculus of the white matter. The lateral dendrites extended to the border of spinal cord where they formed a subpial meshwork (Figure 3), characteristic to the frog spinal cord [8,10]. We used this latter criterion to justify that dendrites were fully labeled and the limb-innervating type of MNs was validated by using the features described above (Figure 3). 

#### Metric morphological description of cervical and lumbar motoneurons

The mean surface area of somata for cervical MNs was about 35% bigger than that of the lumbar MNs but the difference did not reach the significant level because of the high variances in both spinal segments (Table 3, Mann–Whitney-test, p = 0.27). Somata of lumbar MNs were proved to be rounder than those in cervical MNs (Mann–Whitney-test, p < 0.05). The dendrites of lumbar MNs had bigger combined dendritic length, and presented more dendritic branches with a higher maximum branching order (Mann–Whitney test, p < 0.05). The other morphological descriptors did not show statistically significant differences between the dendritic arbors of MNs in the two parts of the spinal cord. In the assessments described above we used pair-wise comparisons with only one descriptor at a time. However, this may be misleading when one compares neurons with many features measured and the aim is their classification. Therefore discriminant analysis using all twelve descriptors of somatodendritic morphology (Table 1) was applied. It classified MNs 100% segmentally correctly (either cervical or lumbar, Wilks’ lambda <0.03, p < 0.007) and the descriptors that segregated MNs significantly were the number of branches (ANOVA, p < 0.001), roundness of somata (ANOVA, p < 0.013) and the combined dendritic length (ANOVA, p < 0.017). In a second analysis we used only six descriptors; those showed significant segmental differences (roundness of somata, number of branches, maximum branching order and combined dendritic length) and the total dendritic surface as well as the maximum distance to endpoints. Discriminant analysis with these descriptors still classified MNs 100% correctly (Wilks’ lambda = 0.18, p < 0.005). To validate these results we used the 16-fold cross validation and the repeated random sub-sampling [40]. Both techniques validated our results on morphological differences of the cervical and lumbar limb moving MNs (average Wilks’ lambda = 0.18, p < 0.005, 87.5% of cross validated grouped cases were correctly classified by 16-fold cross validation; Wilks’ lambda = 0.11, p < 0.05, 77.3% of grouped cases were correctly classified by repeated random sub-sampling). 

**Table 3:** Morphological variables describing the somata, stem dendrites and the dendritic architecture of limb moving motoneurons

**Group**	**Variables**	**Cervical MNs**	**Lumbar MNs**
**Soma**	* Roundness	3.9 ± 0.3 (3.9)	2.6 ± 0.3 (2.8)
	Surface (μm^2^)	9212 ± 1347 (7642)	6776 ± 652 (6885)
**Stem dendrite**	Number	4.8 ± 0.6 (4)	5.7 ± 0.6 (5)
	Sum of diameters (μm)	26.4 ± 1.9 (27.8)	32.7 ± 3.9 (32.1)
**Dendritic tree**	* Total number of branches	127 ± 3.7 (127)	216 ± 5.3 (226)
	* Max. order	9.9 ± 0.6 (9)	11.3 ± 0.4 (11)
	* Combined (total) dendritic length (μm)	32908 ± 3087 (32818)	54522 ± 7083 (60783)
	Surface (μm^2^)	141975 ± 6717 (141491)	193239 ± 18270 (163233)
	Mean parent length (μm)	145.4 ± 2.8 (138.6)	163.7 ± 6 (154.7)
	Mean distance to BRP (μm)	390.7 ± 8.8 (380.3)	495.3 ± 24.2 (448.6)
	Mean distance to ENDP (μm)	881.8 ± 40.1 (831.9)	973.5 ± 114.4 (881.4)
	Max distance of ENDP (μm)	1615.1 ± 100 (1596.3)	2034.5 ± 257.2 (1925.8)

Variables with statistically significant differences between limb moving motoneurons of the two parts of the spinal cord are marked by asterisks (Mann–Whitney-test, p < 0.05). BRP and ENDP are branch point and end point of the dendritic trees. Values are means ± S.E.M.s, medians are in brackets.

#### Distribution of branch points

**Figure 4:**
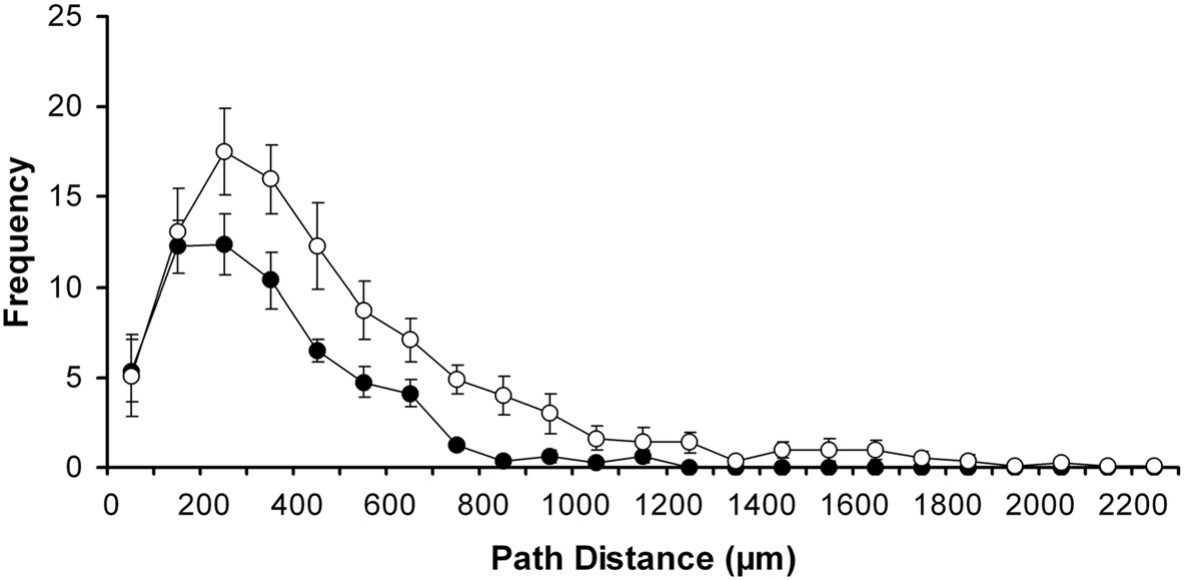
**Spatial distributions of branch points in dendritic trees of limb moving MNs.** Distances of branch points were measured along dendritic paths from the soma. Closed and open circles stand for the cervical and lumbar MNs respectively. Both the mean total numbers and the distributions of branch points are significantly different (Mann–Whitney test, p < 0.005; Wilcoxon test, p < 0.0005) in MNs of the cervical and lumbar parts of the frog spinal cord.

The total number of branch points was more numerous in lumbar MNs than in MNs of the cervical part of the spinal cord (101 ± 18.9 and 59 ± 4.8 in lumbar and cervical MNs respectively, Mann–Whitney test, p < 0.005). The distributions of branch points over different path distance domains were also different (Wilcoxon test, p < 0.0005, Figure 4). In the proximal region (closer than 200 μm to the soma) the numbers of branch points were the same. The biggest difference was found at 200–300 μm distance from the soma, where the distribution curves peaked for MNs of both parts of the spinal cord. Beyond 300 μm, the frequency of branch points showed a steeper decrease with distance in cervical MNs. Dityatev et al. [42] found a similar relation between cervical and lumbar MNs for the probability of bifurcation in the function of branch order. 

We did not find any significant difference in the average lengths of dendritic branches in the proximal region (154 ± 6 μm and 131 ± 3.9 μm in cervical and lumbar MNs respectively, Mann–Whitney-test, p = 0.83). This indicates that difference in branch point frequencies was due to longer total length of dendrites with the same average tendency of branching in the lumbar MNs close to somata as also found by Dityatev et al. [42].

#### Orientation of dendritic trees of motoneurons

Early analyses of dendritic orientation of limb moving MNs in frog spinal cord described three dendritic arrays that extended in the dorso-medial, dorsal and lateral directions [8,43]. Based on the observation that these dendrites with different orientations tend to receive synaptic contacts from different sources, these dendrites were suggested to serve as different input channels to MNs.

**Figure 5:**
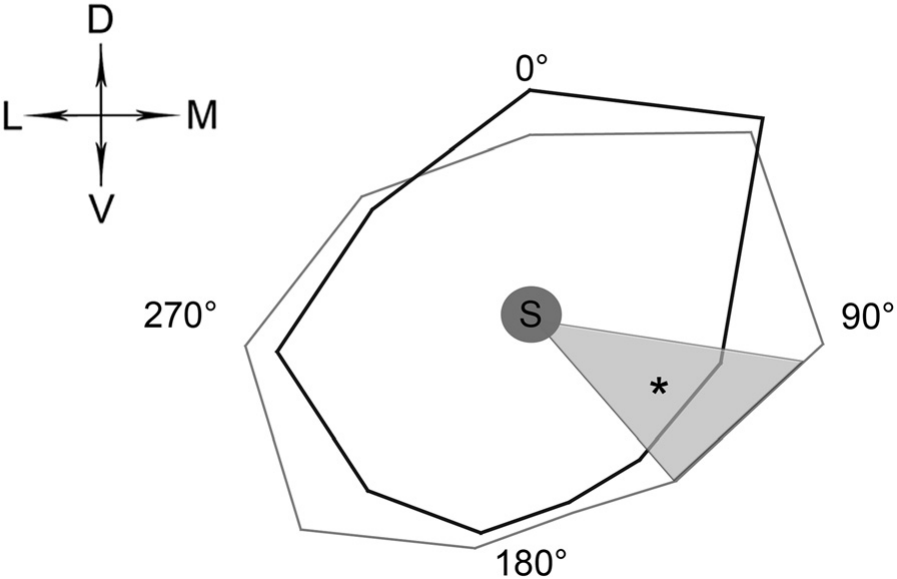
**Polar histograms showing angular distributions of dendritic lengths projected to the transverse plane.** Full circle around somata (S) was divided into 40 degree angle intervals starting with the dorsal direction (0°), the total lengths of dendritic branches were measured within these intervals and averaged over MNs within the same part of the cord. Mean dendritic lengths were represented on a relative scale by the length of a line drawn from the soma in the given direction and finally end points of these lines were interconnected (gray line for the cervical MNs and black line for lumbar MNs). The direction with the longest dendritic length was taken as 100% for the lumbar and cervical MNs separately. Ventromedial (VM) direction (120–160°), where significant segmental difference in angular distributions of dendrites was detected (Mann–Whitney-test, p < 0.05) is shaded in gray.

Here we compared orientation of MN dendrites in the lumbar and cervical levels of the cord. Polar histograms were created (Figure 5) to show average dendritic lengths within equal angle domains around the perikarya of MNs in the transverse plane of the spinal cord. To identify the anatomical directions where lengths of dendritic projections differentiated significantly between cervical and lumbar MNs pair-wise comparisons were used. We found that ventro-medial dendritic extension was significantly bigger in the cervical MNs (Mann–Whitney test, p < 0.05). This is well correlated with the anatomical finding that the ventro-medial region of dendritic trees in cervical MNs is the exclusive terminal zone for tecto-spinal pathways in frogs and this type of connection is not received by lumbar MNs [44]. 

### Morphoelectrotonic transformation of motoneurons

#### Qualitative analysis

**Figure 6:**
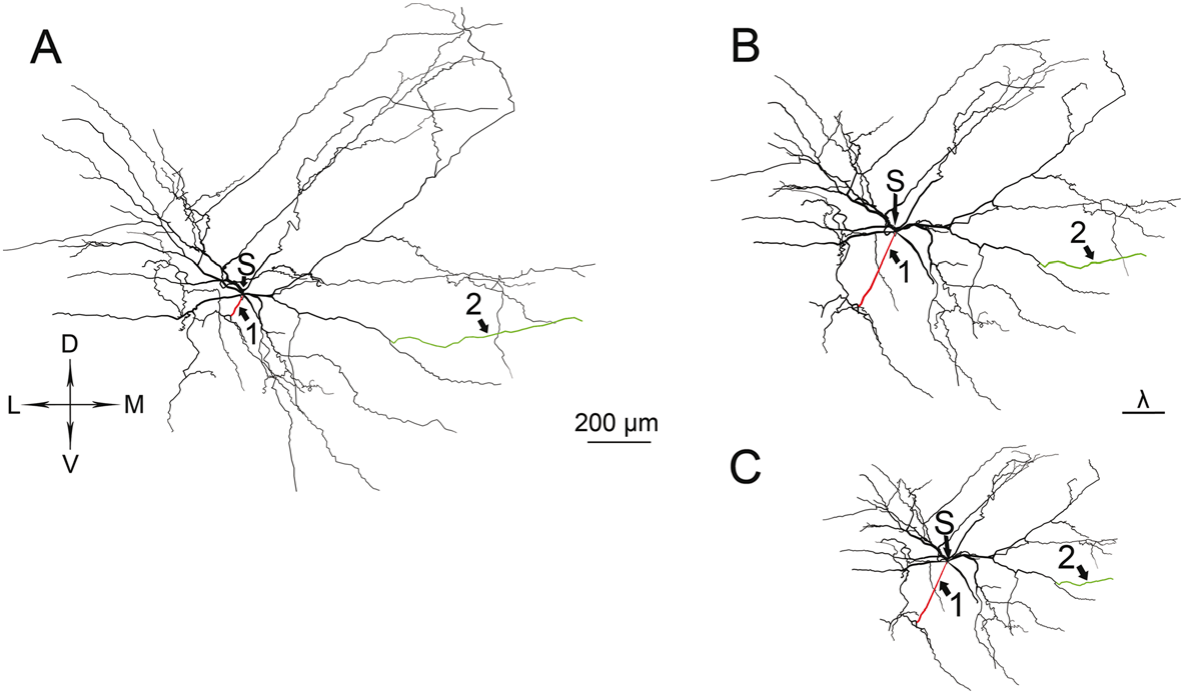
**Dendritic morphology and morphoelectrotonic transforms of a cervical motoneuron.** Morphology (**A**) and METs (**B**) and (**C**) of the same MN (C-167) with homogeneous (R_ms_ = R_md_) and inhomogeneous (R_ms_ < R_md_) soma-dendrite membranes constrained by the physiological 5 MΩ somatic input resistance of the MN for both METs. Arrows 1 and 2 point to homologous proximal (red) and distal (green) dendritic branches where changes during METs are visibly non-proportional to their geometrical sizes (for quantitative analysis see body text). Arrows labeled by S point to the center of the soma (its entire shape is not shown in the figures). D-dorsal, V-ventral, M-medial, L-lateral directions. Note the different size of the MET with our choice of the physiologically constrained pair of Rms-Rmd values in the inhomogeneous soma-dendritic membrane (**C**) relative to the MET of the same MN with homogeneous membrane (**B**) drawn to a common scale of space constants. Both METs show attenuations of somatopetal PSP propagation (‘V_in_ mode’ in NEURON) at DC input (frequency = 0 Hz), recording electrode was at mid-soma, stimulating electrode was at mid-points of dendritic compartments. Dendritic and somatic specific membrane resistances (R_md_ and R_ms_) were equally 8348 Ωcm^2^ for the homogeneous soma-dendritic membrane, while for the MET with inhomogeneous membrane R_md_ and R_ms_ were 20000 and 1046 Ωcm^2^ respectively. With these R_ms_ and R_md_ values the somatic input resistance was 5 MΩ in both membrane models of the MN.

Since it is difficult to infer how MNs’ dendritic architecture affects electrical signal propagation, we used the graphical approach of the morphoelectrotonic transformation (MET, [35]). By this method it was possible to analyze the somatopetally propagating PSPs and to relate their rates of attenuations to the geometry of dendrites. We illustrated this relationship in Figure 6, where part A shows the geometrical structure of an individual cervical motoneuron (C-167), while parts B-C show two METs of the same MN. The MET maps the anatomical architecture of the dendrites to electrotonic space using the log attenuation of PSPs as the distance metric keeping the original topology and branching angles allowing visual comparison of morphology and signal propagation in dendrites. Comparisons are easy because the distances in the METs are proportional to the logarithm of the ratio of time integrals of the voltage responses at any two points, the metric used to measure attenuations of PSPs [35]. Beside the dendritic geometry, the MET is also dependent on the biophysical properties of neurons. Therefore, four METs were created for each cervical and lumbar MN. We studied the METs of MNs with physiologically constrained homogeneous (R_ms_ = R_md_) and inhomogeneous (R_ms_ < R_md_) soma-dendritic membranes (see Methods) at different neuron resistances. 

If the METs of the same MN with homogeneous and inhomogeneous soma-dendrite membranes but with identical somatic neuron resistances are compared the size (compactness) of the MET may be different (Figure 6B–C) indicating different rates of attenuations of PSPs along the dendrites due to changes in leakiness of dendrites (R_md_) and the soma (R_ms_). Note that both R_md_ and R_ms_ are different in these two METs; R_md_ is bigger, while R_ms_ is smaller in Figure 6C. An increase in R_md_ makes the MET more compact, while the decreasing R_ms_ has the opposite effect on compactness. When the geometry and the METs of dendritic arbors were compared, careful inspection found non-proportional changes in the MET size of dendritic branches relative to their morphological appearance (see branches 1 and 2 in Figure 6).

#### Quantitative analysis of morphoelectrotonic transforms

##### Comparison of different morphoelectrotonic transforms

To compare different METs of dendrites (representing electrotonic architectures in different membrane models) of the same MN, a set of MET descriptors (Table 2) was computed. These descriptors were in analogy with those used in metric morphological description of the dendritic trees (see Tables 1 and 3) but geometrical distance was replaced by electrotonic length here. Significant differences were found in combined MET lengths (p < 0.05, Mann–Whitney test) between the cervical and lumbar MNs in all models irrespective of the inhomogeneity of the membrane and the neuron resistance. When the neurons’ input resistance was 5 MΩ and the membrane was inhomogeneous (R_ms_ < R_md_), cervical and lumbar MNs also differed in their mean MET distances measured to branch points.

Discriminant analysis using MET descriptors classified cervical and lumbar MNs 100% and 95% correctly in all membrane models (Wilks’ lambda <0.02, p < 0.005). Similar results were obtained when the number of descriptors was reduced. With just four descriptors (combined MET length, mean MET distance to branch points, mean MET distance to end points and the maximum MET distance to end points) MNs were still classified 87% segmentally correctly. These results were cross validated by the 16-fold and the repeated random subsampling techniques [40] (in average more than 70% of cross validated grouped cases were correctly classified with an average Wilks’ lambda of 0.25 and p < 0.05).

##### Comparison of morphoelectrotonic transforms of dendrites with their original morphology

In case of the two previously selected dendritic branches we calculated their geometrical and MET size ratios. The lengths of the proximal and distal branches were 84 and 533 μm respectively (branch 1 and 2 in Figure 6). The log attenuations along these branches (their sizes in the METs) were 1.97 and 2.50 λ in the MET with homogeneous soma-dendritic membrane and 1.94 and 1.68 λ in the MET with inhomogeneous (R_ms_ < R_md_) membrane. These values yield 84/533 = 0.16 proximal to distal ratio for the geometrical size of branches, and 1.97/2.50 = 0.79 and 1.94/1.68 = 1.15 ratios in the METs with homogeneous and inhomogeneous membranes respectively. This observation suggests non-proportional changes in the size of dendrites during the MET, which depend on the distance of dendritic branch from the soma.

**Figure 7:**
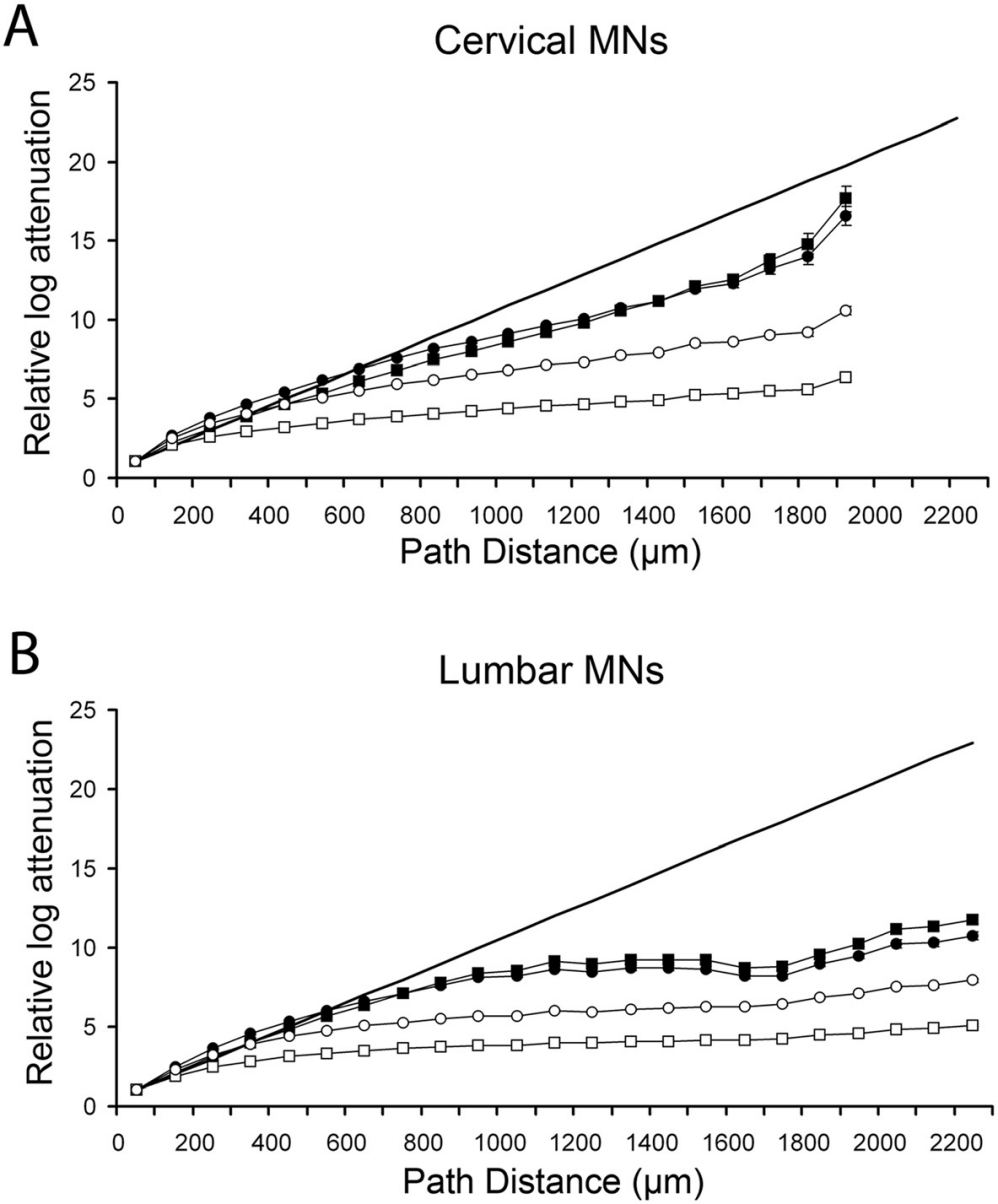
**Comparison of morphoelectrotonic transformations with their original geometry in (A) cervical and (B) lumbar MNs.** Somatopetal log attenuations of PSPs were computed from thousands of dendritic locations per neuron and divided by the mean attenuation calculated from locations within 100 μm from the soma. Finally, these ratios (relative log attenuations) were averaged and graphed over 100 μm path distance ranges from the soma. Attenuation ratios were computed in four different models of MNs by using 1.4 MΩ neuron resistance with homogeneous (R_ms_ = R_md_) and inhomogeneous (R_ms_ < R_md_) soma-dendritic membranes (closed and open rectangles) and by 5 MΩ neuron resistance with homogeneous and inhomogeneous membranes (closed and open circles). In homogeneous membrane models R_md_ was equal to R_ms_, in inhomogeneous models R_md_ = 20000 Ωcm^2^ was assumed. The common specific membrane resistance for the soma and dendrites in homogeneous models and the R_ms_ values in inhomogeneous models were defined to have neurons with 1.4 or 5 MΩ input resistance measured at the soma. Continuous linear thick line is a reference where data points would be positioned if METs cause proportional changes in size of dendrites relative to their morphological appearance. Note that many error bars, representing S.E.M.s, are too small to be visible because of the high numbers of sampling sites.

To investigate these changes further, log attenuations of PSPs to soma were determined from different regions of the dendrites located within 100 μm path distance domains from the perikaryon. Then, the computed attenuations were divided by the mean attenuation of PSPs measured from dendritic sites within 0–100 μm from the perikaryon. These relative attenuations were not linearly related to the path distances of locations where PSPs were generated indicating again a non-proportional change in size of dendrites during the METs (Figure 7). Relative log attenuations deviated more from the linear reference line in the distant regions and the size of this deviation depended on the membrane model used. Rates of log attenuations computed in a given membrane model were proved to be different in cervical (Figure 7A) and lumbar (Figure 7B) MNs. In MNs from the cervical spinal cord, the METs caused more proportional changes in the size of dendrites (data points were closer to the reference line), while in lumbar MNs the ratios of attenuations showed increasingly bigger deviations from the reference line in the more distant domains (Figure 7, see also dendrites marked by arrows in Figure 6). The biggest deviations from the proportional change were found in METs when inhomogeneous (R_ms_ < R_md_) membrane model with 1.4 MΩ neuron input resistance was assumed.

##### Rates of log attenuations are different in cervical and lumbar motoneurons

**Figure 8:**
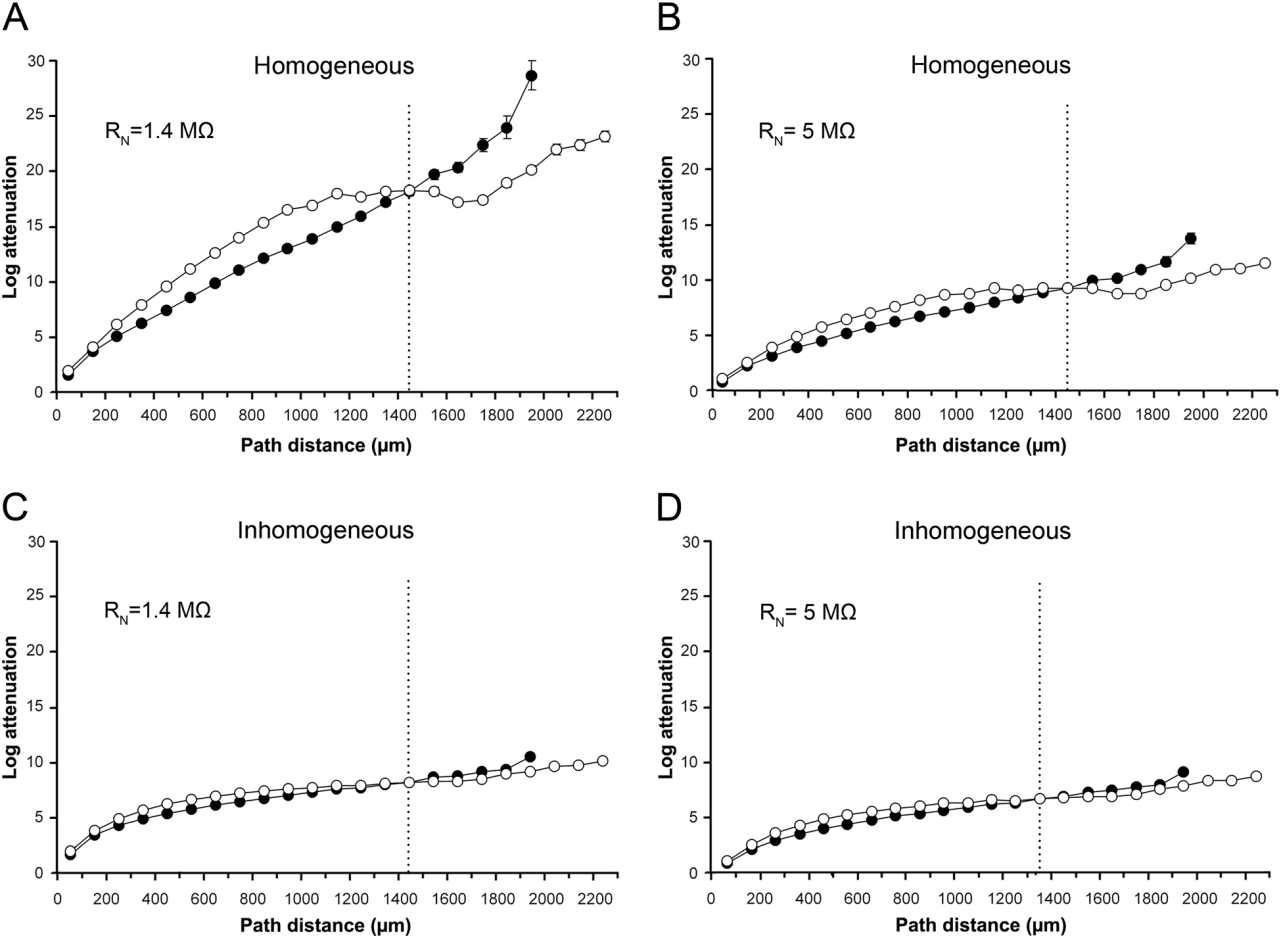
**Comparison of somatopetal log attenuations of postsynaptic potentials.** Attenuations of PSPs were computed from dendritic locations and averaged within 100 μm path distance ranges from the soma. Homogeneous (R_ms_ = R_md_, part **A** and **B**) and inhomogeneous (R_ms_ < R_md_, part **C** and **D**) soma-dendrite membrane models were used with 1.4 MΩ (**A** and **C**) and 5.0 MΩ (**B** and **D**) neuron resistances, where R_ms_ and R_md_ values were defined as described in the legend of Figure 7. A characteristic 1400–1500 μm distance, the limit of distance domains where attenuations in cervical (closed circles) and lumbar MNs (open circles) were significantly different were marked by vertical dotted lines. Many error bars are not visible due to their very low values.

The mean somatopetal log attenuations of PSPs were determined as a function of path distance from the soma in different membrane models of the cervical and lumbar MNs (Figure 8). Generally, PSPs attenuated differently (Mann–Whitney test, p < 10^−6^) from the same geometrical distance in lumbar and in cervical MNs both in the homogeneous (R_ms_ = R_md_), Figure 8A–B) and in the inhomogeneous (R_ms_ < R_md_, Figure 8C–D) soma-dendritic membrane models. If the distance of the site of PSP initiation was closer than 1400–1500 μm to the soma then PSPs attenuated more in the lumbar MNs, while PSPs generated farther than this characteristic distance attenuated less in lumbar MNs. The characteristic distance separating the two distance domains from where PSPs attenuated differently was virtually independent of the size of inhomogeneity of the soma-dendritic membrane and the input resistance of neurons. Differences between the rates of log attenuations in cervical and lumbar MNs were reduced when inhomogeneous rather than homogeneous soma-dendritic membrane was assumed. However, even small but significant differences measured on a logarithmic scale by log attenuations may be translated to considerably divergent voltage-time integrals on the somata of segmentally different MNs.

### Structural comparison of signal transfer properties

Nerve cells with significantly different sizes are likely to be different electrotonically too. However, it remains an important question if neurons with different sizes keep their electrotonic difference if their comparison is based only on their topological structure and size-dependency is ignored. Here we studied this issue by using size-independent comparisons of dendritic signal transfer properties in cervical and lumbar MNs. This type of electrotonic comparison is feasible since electrotonic structure - in analogy to the geometrical structure - is not only defined by metric-related properties, but also by the branching structure (topology) of dendritic trees affecting the shape of distributions of voltage and current transfer properties over the length of dendrites.

**Figure 9:**
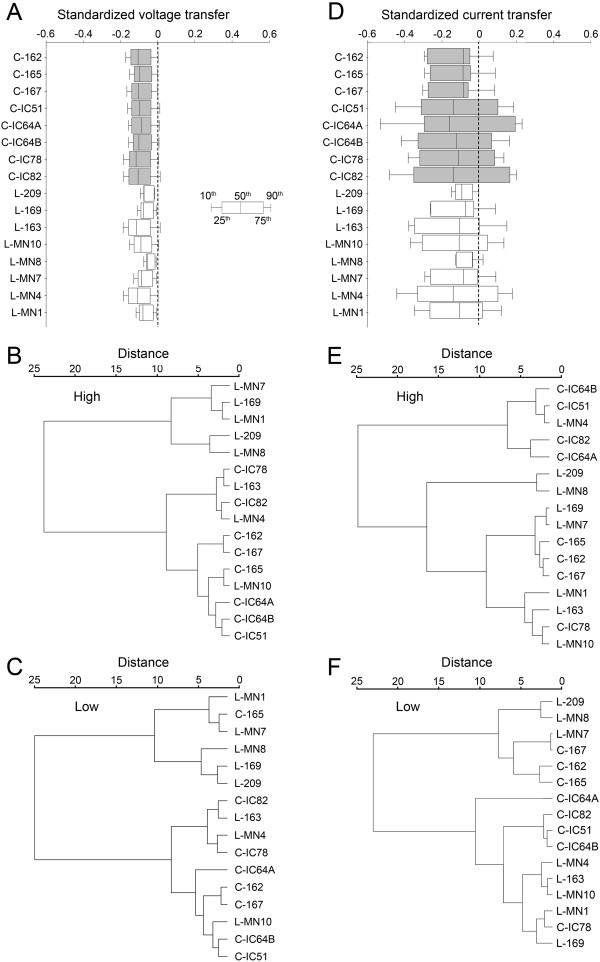
**Voltage and current transfers in steady-state.** Box plots show 10^th^, 25^th^, 50^th^, 75^th^ and 90^th^ percentiles of standardized and area weighted voltage- (**A**) and current transfers (**D**) in steady-state measured from dendritic points to the soma in limb moving MNs in the cervical (shaded boxes) and lumbar (open boxes) segments. Higher and lower percentiles were weighted relative to the median (50^th^ percentile) and used as descriptors for cluster analysis of MNs. The 10^th^ and 90^th^ as well as the 25^th^ and 75^th^ percentiles are shown by the wings and by the borders of boxes respectively, the median is marked by the line within the box (see insert). Dendrograms show segmental segregation tendencies between the cervical and lumbar MNs based on voltage and current transfer properties under high (voltage: part **B**, current: part **E**) and low (voltage: part **C**, current: part **F**) background synaptic activities. Different levels of synaptic background activities were modeled by varying the specific dendritic membrane resistance (R_md_). High activity: R_md_ = 5000 Ωcm^2^ and low activity: R_md_ = 50000 Ωcm^2^. In cluster analyses shown, the Ward’s method was used and the weighting factors for percentiles were: 0.2 for the 10^th^ and 90^th^ percentiles and 0.8 for the 25^th^ and 75^th^ percentiles. MN labels starting with letters C and L stand for the cervical and lumbar neurons respectively.

**Figure 10:**
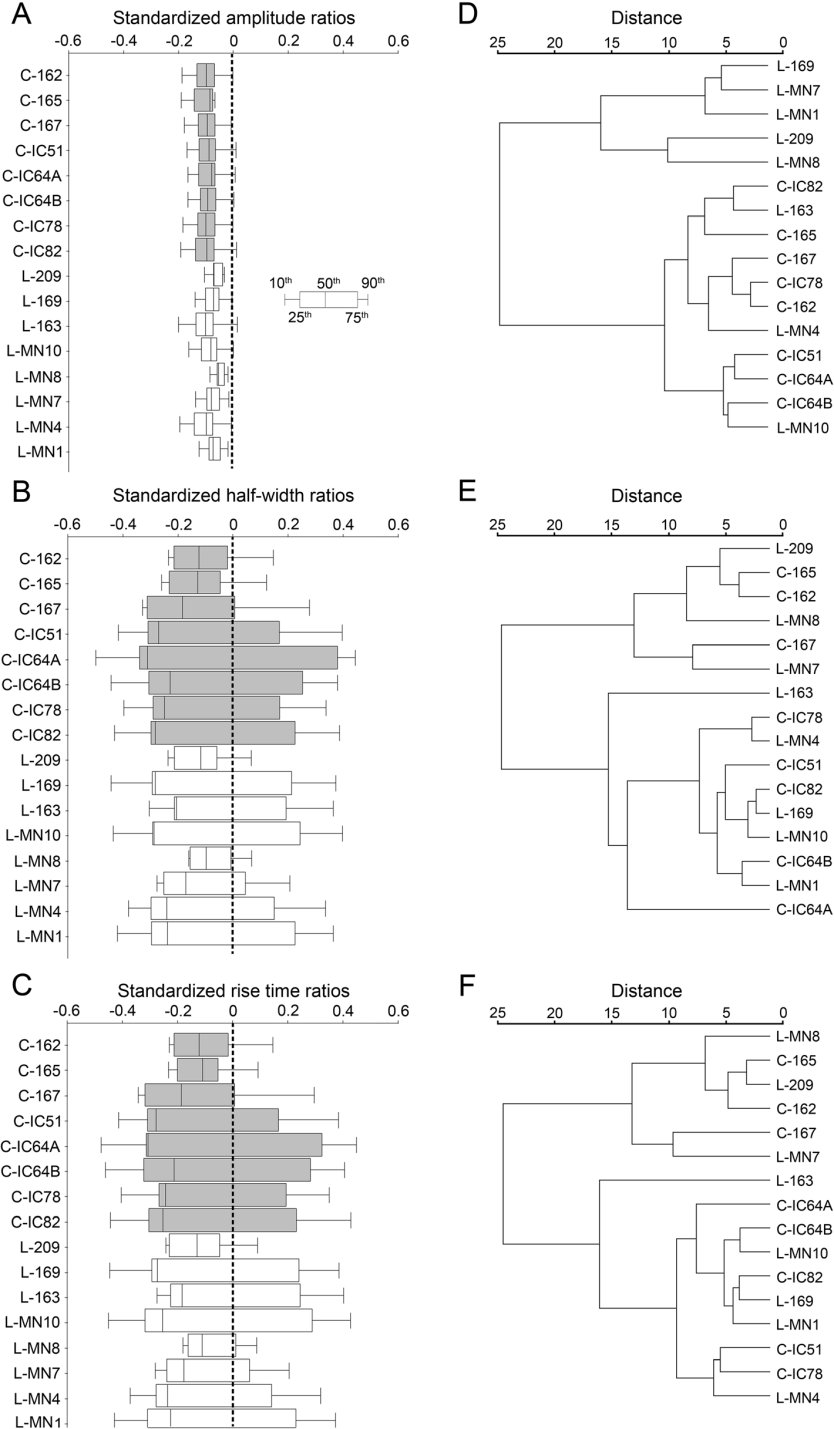
**Propagation of voltage transients.** Somatic to dendritic ratios of PSP amplitudes, half-widths and rise times were computed for each dendritic compartment, ratios were then weighted by the area of the compartments and distributions were standardized. Percentiles are shown as box plots (see insert) for amplitudes (**A**), half-widths (**B**) and rise times (**C**) respectively (boxes for cervical MNs are shaded). These percentiles were then weighted relative to the median and used as descriptors in cluster analysis. Dendrograms of cluster formations (using Pair group method) were based on ratios of amplitudes (**D**), half-widths (**E**) and rise times (**F**). A control, or middle level of synaptic background activity (R_md_ = 20000 Ωcm^2^) was assumed in all cases shown. Labels starting with letters L and C stand for cervical and lumbar MNs respectively. The weighting factors for percentiles were: 0.33 for the 10^th^ and 90^th^ percentiles and 0.67 for the 25^th^ and 75^th^ percentiles.

To focus on such structural rather than size-related features of dendrites, we used distributions of standardized and area weighted voltage and current transfer values [34]. The data processing is exemplified in Figure 1 by the steady-state currents transfers. The starting point is the set of transfer values computed between the mid-points of each dendritic compartment and the soma. The frequency distribution of these transfers (Figure 1A) gives equal weight to each measurement (compartment). However, transfers measured from larger compartments approximate attenuations for more synapses since the number of synapses received by a dendritic compartment is directly proportional to the area of the compartment [37]. To take this into account, in the second step, the raw measurements of transfers were area weighted (Figure 1B) to give proportionally bigger weight to compartments with larger surface area. Finally, area weighted distributions of signal transfers were standardized (Figure 1C) to create distributions with shapes, characteristic to signal transfer properties of neurons independently of their variable size. The shapes of these standardized distributions were described by their 10^th^, 25^th^, 50^th^, 75^th^ and 90^th^ percentiles (these were graphed as box plots in Figures 9 and 10). The percentiles were then weighted relative to the median and used as descriptors of the standardized distributions in the cluster analysis to reveal grouping tendencies of cervical and lumbar MNs. While cluster analysis is generally considered as an objective way to reveal grouping of objects, the method suffers from the lack of criteria on how the similarity level should be chosen where cluster formations are analyzed. Here, we decided to study cluster formations at the end of the hierarchical cluster analysis, when the two biggest clusters appear before including all MNs in a single group (last order clustering, Figure 2, see also Methods). The advantage of this method was the determination of the similarity level by the dendrograms themselves and not by the investigator who carried out the analysis. 

Segmental segregation was measured by homogeneity indexes of the last order clusters. The significance of segmental segregation was tested by comparing these homogeneity indexes with those calculated for clusters formed when segmental origin of MNs was randomized (see Methods for more details).

#### Steady-state signal transfer

In this set of analysis a constant current was injected to generate steady depolarizations in the midpoints of all cylindrical compartments of the cable model and voltage and current transfers to soma were measured. These transfer values were then processed as summarized above.

##### Voltage transfer

**Figure 11:**
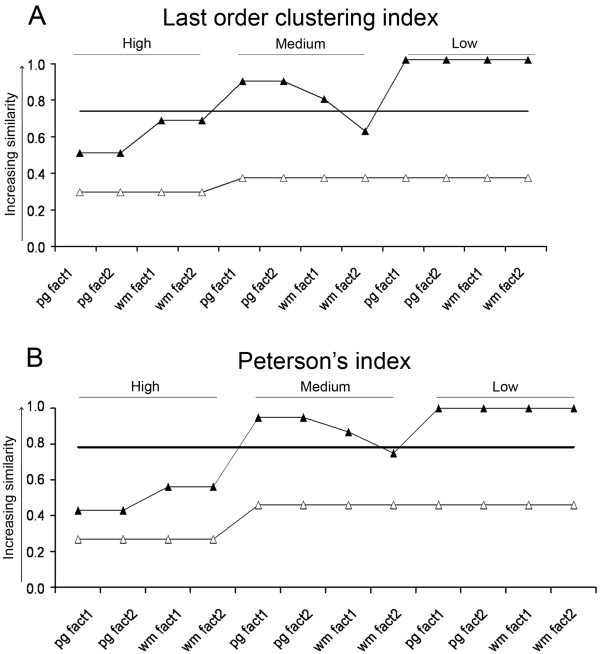
**Grouping tendencies of MNs based on steady-state voltage- and current transfers (open and closed triangles).** ‘High’, ‘Medium’, and ‘Low’ levels of synaptic background activities on dendrites were modeled by 5000, 20000 and 50000 Ωcm^2^ specific dendritic membrane resistivities respectively. To reveal grouping tendencies cluster analysis was used with the Pair group and Ward’s methods (see horizontal labels starting with ‘pg’ and ‘wm’) with differently weighted (‘fact1’ and ‘fact2’) descriptors. The five descriptors were the 10^th^, 25^th^, 50^th^, 75^th^, and 90^th^ percentiles of standardized and area weighted distributions of voltage and current transfers between dendritic points and the soma. The two sets of weighting factors of percentiles (‘fact1’ and ‘fact2’) were as follows: In factor set 1, the 10^th^ and 90^th^ percentiles were weighted by 0.2 and the 25^th^ and 75^th^ percentiles by 0.8. In factor set 2, the weighting factors were 0.33 for the 10^th^ and 90^th^ percentiles and 0.67 for the 25^th^ and 75^th^ percentiles. In both sets of weighting factors the weight was 1 for the 50^th^ percentile. In cluster analyses the Euclidian distances were used. Homogeneity indexes, last order clustering index (**A**) and Peterson’s index (**B**), were used to measure segmental homogeneities of MNs within last order clusters, which reflect segregation of cervical and lumbar MNs between the clusters. Homogeneity indexes with values closer to one indicate higher similarity (poorer segregation) of cervical and lumbar MNs. Continuous horizontal lines mark the levels of homogeneity indexes below which segmental separation of MNs by their voltage and current transfer properties is significant.

By using somatopetal voltage transfers, our first observation was the higher variabilities for all, except the 90^th^ percentiles of distributions for the lumbar MNs (F-test, p < 0.02) and the generally shifted nature of percentiles relative to those of the cervical MN group (Figure 9A). Indeed, hierarchical cluster analysis proved segregation tendency of the cervical and lumbar MNs. Under high background synaptic activity one of the last order clusters was homogeneous and contained five lumbar MNs without any cervical ones, while the other cluster contained all cervical neurons along with three lumbar ones (Figure 9B). The tendency of segregation was present at all intensities of synaptic background activity but segregation was a bit weaker if synaptic activity was low (Figure 9C). In this case the two last order clusters contained lumbar and cervical MNs in ratios of 5:1 and 3:7. The significance in the tendency of segmental segregation was tested by comparing the actual homogeneity indexes of last order clusters (last order clustering index and Peterson’s index) to the mean indexes calculated for clusters formed when segmental origins of MNs were artificially randomized. In all of these comparisons, indexes remained below their critical values and cervical and lumbar MNs were proved to be segmentally different (Figure 11A, B, one sample *t*-test, p < 10^−18^) in their steady-state voltage transfer properties. These segmental differences between MNs were detected at all levels of background synaptic activities with some tendency of MNs to get more similar with the decrease of background activity.

##### Current transfer

When signal transfer properties of MNs were characterized by current transfers we found, similarly to the voltage transfer, that lumbar MNs showed higher variabilities in their medians (F-test, p < 0.03) with a less obvious general shift in the standardized distributions (Figure 9D). Cluster analysis showed a segregation of lumbar and cervical MNs in 1:4 and 7:4 ratios in the last order clusters at high background synaptic activity (Figure 9E), but there was no segregation if synaptic activity was low (Figure 9F). In this latter case last order clusters contained MNs of the two segments in equal numbers. Analysis of homogeneity indexes corresponded to these observations and significant segmental segregation among MNs was found only at high synaptic background activities (one sample *t*-test, p < 0.02). MNs were getting more and more similar with the decrease of overall synaptic activity (Figure 9E, F and Figure 11A, B), as we found when MNs were characterized by voltage transfers. The trend of increasing similarities of cervical and lumbar MNs with the decrease of background synaptic activity was obvious by both similarity indexes and was independent of the cluster analysis and weighting techniques (Figure 11).

#### Transient signal transfer

While steady-state approach measures transfer properties when the generation of PSPs can be approximated by a constant current injection, many synaptic events are short in time and are better approximated by transient conductance changes. Propagation properties of these transient voltage signals are different than those under steady-state circumstances. Therefore, we extended our analysis and investigated transfers of voltage transients. In these simulations PSPs were generated by brief conductance changes in dendritic points. The changes in shape parameters (amplitude, half-width and rise time) of transient PSPs were computed during their propagation to the soma and descriptors of these changes were created as described earlier. Box plots of these descriptors, once again, showed that lumbar MNs were more variable than the cervical ones in the way the amplitudes of voltage transients were reduced during their propagation to the soma (Figure 10A, F-test, p < 0.003). However, variabilities in changes of the somatic to dendritic ratios of half-widths and rise times were similar in the MNs of the two segments (F-test, p > 0.05, Figure 10B–C).

##### Cluster formations based on attenuation of peak potentials

**Figure 12:**
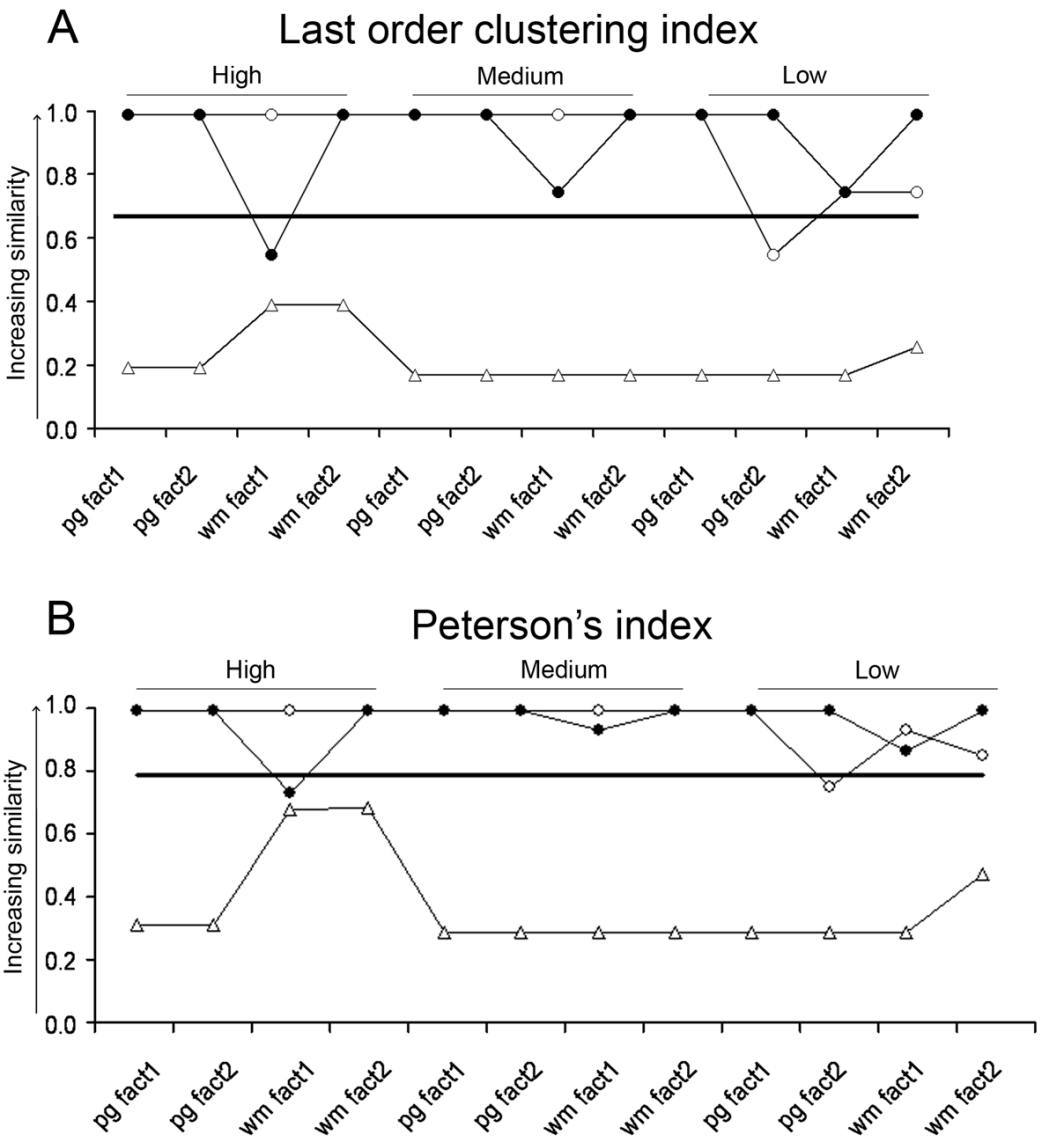
**Segregation of cervical and lumbar limb moving motoneurons based on somatopetal propagation of voltage transients.** Cluster analysis was used with the Pair group and Ward’s methods (see horizontal labels starting with ‘pg’ and ‘wm’) with differently weighted (‘fact1’ and ‘fact2’) descriptors. These descriptors were the standardized and area weighted percentiles of somatic to dendritic ratios of peak potentials (open triangles), half-widths (closed circles) and rise times (open circles) of PSPs to quantify the changes in shape of voltage transients generated by conductance changes according to an α-function (g_max_ = 2 nS, t_max_ = 1.5 ms). Last order clustering index (**A**) and Peterson’s homogeneity index (**B**) were used to measure homogeneities within last order clusters, which reflect segregation of cervical and lumbar MNs between these clusters. Homogeneity indexes with values closer to one indicate higher similarity (poorer segregation) of cervical and lumbar MNs. Continuous horizontal lines mark levels of homogeneities below which separation of MNs is significant. ‘High’, ‘Medium’, and ‘Low’ levels of synaptic background activities on dendrites were modeled by 5000, 20000 and 50000 Ωcm^2^ specific dendritic membrane resistivities respectively.

The two last order clusters showed significant segmental homogeneity in the origin of MNs they contained (Figure 10D) when cluster analysis was based on somatic to dendritic amplitudes of voltage transients. One of the last order clusters was purely homogeneous and contained five lumbar MNs while the other accommodated cervical and lumbar MNs in 8:3 ratio. The homogeneity indexes of these last order clusters were significantly lower (one sample *t*-test, p < 0.0005) than those calculated for clusters when segmental origin of MNs was artificially randomized (open triangles in Figure 12A, B). Consequently, there was a significant segmental segregation tendency between the cervical and lumbar MNs based on the attenuation of transient EPSP amplitudes. This tendency was present at all intensities of synaptic background activity.

##### Cluster formations based on somatic to dendritic ratios of half-widths and rise times

Neither the box plots (Figure 10B, C) nor the last order clusters (Figure 10E, F) showed any segmentum-wise segregation of the MNs based on changes of half-widths and rise times of voltage transients. Last order clusters contained equal numbers of MNs from both spinal segments (Figure 10E, F). Therefore, as expected, segmental homogeneity of the two last order clusters did not differ significantly (one sample *t*-test, p > 0.96) in these cases. Graphs of both homogeneity indexes tended to remain in the territory (above their critical values) where they represent no significant segregation of the two groups of MNs. (Figure 12A, B).

The results obtained on propagation of voltage transients may be summarized in the following way: 1) Cervical and lumbar limb-moving MNs of frogs have structurally different dendrites imposing different attenuations of voltage amplitudes during their propagation to the soma. 2) On the other hand, these structural differences in dendrites do not distinguish the two classes of MNs in the way how half-widths and rise times of transient potentials are changing during their dendritic propagation.

## Discussion

We investigated morphological and electrical differences between cervical and lumbar spinal motoneurons (MNs) that innervate fore- and hindlimb muscles in adult frogs. We deliberately did not want to compare MNs according to the specific muscles they innervate [4,45-48] rather we looked for their properties that are distinct in the cervical and lumbar segments for the following reasons.

MNs undergo substantial developmental changes during embryological and postnatal life that affect the size of cell bodies, size and branching structure of dendrites and these changes are accompanied by physiological maturation of membrane properties like specific membrane resistance, neuron resistance, resting membrane potential, spike shapes and excitability [49]. Some of these changes have been shown to occur in a rostro-caudal sequence along the neural axis [50]. Recent evidence suggests that MN pool identity and nerve trajectories are, at least to some extent, predefined genetically. The location-specific expression of Homeobox (Hox) genes along the rostro-caudal axis controls the emergence of lateral motor column at the brachial and lumbar levels in the chick and mouse [51-53]. The Hox genes also regulate intrasegmental MN specification and their target muscle connectivity [51]. In the frog, the Hoxc6 gene has also been shown to affect primary neurogenesis [54].

Overall, these findings suggest that MN maturation is highly dependent on rostro-caudal position of neurons and therefore intrinsic differences in morphology and electrical properties of MNs may be expected to occur along the rostro-caudal axis. These differences have been investigated in the present study.

### Methodology

#### Choice of statistical methods

To investigate segmental differences between MNs, we used pair-wise comparisons of morophological and electrotonic properties, multivariate discriminant analysis and cluster analysis. These methods have been successfully applied in classifying spinal MNs in the turtle [55], tectal efferent neurons in monkeys [56], MNs of the jaw-closing and opening muscles in the brainstem [57], MNs involved in tongue movements in adult frogs [58] and spinal interneurons in frog embryos [59]. A new element in our method was that we combined cluster analysis with application of homogeneity indexes to investigate the grouping tendencies of limb innervating MNs based on their segmental origin. Cluster analysis involves establishment of an artificially chosen similarity level where cluster formations are analyzed. However, in cases where the significance of grouping tendencies cannot be judged visually, a less artificial establishment of the similarity level and a more rigorous analysis of the clusters formed at that level are needed. To carry out such an analysis we set the similarity level in a way that was defined by the dendrogram itself and we analyzed segmental homogeneity of clusters formed at this level (last order clustering) by applying the concept of homogeneity indexes, which are widely used in ecology e.g. to measure species homogeneity or diversity in territorially different sampling sites [60-62]. We used two different homogeneity indexes to measure segmental homogeneities in the two last order clusters. In our case, higher segmental segregation of MNs results in more homogeneous clusters. Whether or not the homogeneity of the last order clusters is higher than expected where no significant segmental separation tendency exists was checked by comparing the homogeneity indexes of actual clusters to those calculated when segmental origin of the same set of MNs was randomly redistributed. When homogeneity indexes of the actual last order clusters indicated significantly higher segmental homogeneities than in the segmentally randomized sample we considered MNs of the cervical and lumbar segments different.

#### Choice of neuron models

We used high-fidelity compartmental cable models with a set of different membrane properties to account for the variability in neuron resistances measured experimentally, to analyze the effects of the varying size of inhomogeneity in the soma-dendritic membrane and to mimic synaptic background activity. However, in our models we considered a passive membrane. This restriction is validated by a number of factors. Although there is an ever growing list of evidence that voltage-dependent ion channels are present in the dendritic membranes of different nerve cells (see [63] for a review), the existing literature evidence is not consistent and therefore it is not easy to estimate the impact of possible active dendritic processes on the current and voltage transfer profiles of dendrites in spinal MNs. Larkum et al. [64] found that experimentally observed attenuation ratios in dendrites could adequately be explained by passive membrane properties used in compartmental modeling to simulate dendritic impulse propagation in the reconstructed geometry of the MN recorded. On the other hand, Czeh [65] predicted presence of voltage-dependent conductances in spinal MN dendrites of frogs based on extracellular recordings.

Subsequent physiological studies suggested involvement of persistent inward currents mediated by voltage-dependent L-type calcium channels of MNs [66-68] in maintenance of limb posture [69] and in production of withdrawal reflexes in the frog [70]. The distribution of Ca_v1.3_ channel, a subtype of L-type calcium channels, was studied immunohistochemically in MNs of the mouse, cat and turtle, but not in the frog [71]. These studies showed essential species differences in the somato-dendritic distribution of Ca_v1.3_ channel [72]. Bistable behavior of the membrane, a major consequence of the persistent inward current through the L-type calcium channels, was suggested to be a characteristic of MNs that innervate fatigue resistant muscles and therefore primarily involved in posture [73] but may not have significant contribution to production of locomotor behavior [70].

The presence of non-linear processes on limb moving MNs in the frog does not rule out the importance of the proper description of passive signal transfer properties of these neurons as a necessary step to be able to elucidate the functional relevance of active channels better. The view that it is much more difficult to understand the influence of voltage-dependent channels in the absence of detailed knowledge on current and voltage transfers imposed by the passive membrane is shared by many neurobiologists [1,33,35,74,75].

### Morphology

In the present study we characterized forelimb and hindlimb moving MNs of the frog with the aid of quantitative morphological parameters that describe the somata, stem dendrites and the rest of dendritic trees. Pair-wise comparisons of the individual variables indicated that lumbar MNs had rounder somata and bigger dendritic trees comprising more dendritic branches than the cervical MNs. Multivariate discriminant analysis could separate cervical and lumbar MNs into two distinct groups according to their somato-dendritic morphology.

#### Accuracy of the reconstructed dendritic diameters

We used state of the art neuron reconstruction systems to digitize the 3D geometry of dendrites. However, the morphological data, as in case of any measurement, cannot be free of errors. One critical parameter is the accuracy of dendritic diameters since they affect dendritic impulse propagation [29,76] and the surface area of dendritic compartments what we used as weighting factors to analyze signal transfer properties of dendrites. The accuracy of tracing of dendritic diameters is limited by the resolution capacity of the reconstruction system. Diameters were traced to 0.5 μm accuracy in ten out of the sixteen MNs reconstructed. Additional three cervical and three lumbar MNs were traced to a higher, 0.1 μm precision using a newer and more accurate version of the Neurolucida (Microbrightfield, USA) reconstruction system. When dendrites were traced with 0.5 μm resolution, the diameters of the smallest calibre (<0.5 μm) dendrites were overestimated since all these diameters were recorded as 0.5 μm, the smallest diameter recordable by the reconstruction system. To estimate the extent and impact of such diameter overestimation we calculated the percentage of dendritic surface given by dendrites with diameters smaller than 0.5 μm in the six MNs where diameters were recorded in 0.1 μm steps. These calculations showed that, in average, only 2% and 3% of the total dendritic surface area was given by thinner than 0.5 μm dendrites in the cervical and lumbar MNs respectively. The minimum dendritic diameter was 0.3 μm and these extremely thin dendrites were always located at a large distance from the soma. From functional point of view, the sensitivity of current transfers to changes in the diameter of small calibre dendrites with distal position from the soma has been investigated in segmental cable models of MNs, where dendritic diameters were artificially varied and current transfers from dendrites to the soma were computed. The changes in current transfer values were less than 1% if thickness was altered by 0.1–0.2 μm in the thin and distal dendrites [29].

Based on the small contribution of the thinnest (<0.5 μm) dendrites to total surface area and the low sensitivity of dendritic impulse propagation to the thickness of these dendrites we conclude, that the impact of 0.1–0.2 μm overestimation of diameters for the thinnest dendrites is unlikely to be significant in our study.

#### Branching structure

We found segmental differences both in the numbers and distributions of branch points in dendrites of limb moving MNs of the cervical and lumbar segments. The bigger number of branch points in lumbar MNs does not merely mean more dendritic branches but also a topologically and electrotonically more complex dendritic architecture. Based on various morphological measures of dendritic complexity Dityatev et al. [42] found that lumbar MNs were more complex than cervical MNs in frogs. Although this difference was not generally significant it was consistent in all complexity measures used. Complexity of MN dendritic trees was correlated with the contractile properties of the muscles they innervated in cats and rats [77] and also in frogs [42]. These authors reported that MNs innervating fast muscles were topologically more complex than those innervating slow muscles. This way, the more complex branching structure of lumbar MNs is correlating well with the faster contractions needed in muscles of the hindlimbs of frogs during jumping and swimming.

Locations of branch points are related to signal propagation in the dendrites both in the presence and absence of voltage–dependent ion channels. In dendrites with passive membrane, the current transfer effectiveness generally changes abruptly at branch points altering the ‘cost’ of moving a synapse to a geometrically different location in terms of the change in the soma potential during synaptic activity [17,78]. In dendrites with active membranes, the backpropagation of action potentials and firing properties have been shown to be dependent on the distribution of membrane surface over the dendrites, which is highly affected by the branching pattern [79,80].

#### Projections of dendrites

Comparison of the orientation of dendritic arborization also demonstrated differences between cervical and lumbar MNs. We found that the ventromedial extension of dendritic trees is more powerful in the cervical MNs. Experiments using retrograde cell degeneration technique showed that the ventromedial area in the gray matter of the cervical segments corresponds to the terminal fields of contralateral tectospinal pathways that do not extend to lumbar segments in frogs [44]. Axon terminals from the lateral vestibular nucleus were also more numerous in the ventromedial area of cervical spinal segments [81]. These data suggest that tectospinal and vestibulospinal pathways may control the function of cervical MNs via this well developed ventromedial dendritic array. Physiological experiments supported that axons from the lateral vestibular nucleus influence cervical MNs [82] and the pathway from the tectum also produced disynaptic excitation and inhibition of forelimb MNs [83]. These descending pathways may be involved in visually guided prey-catching behavior and control the orientation of the head with respect to the prey. In order to keep the prey in the visual filed frogs have to turn their head and these movements are accompanied by movements of the upper limbs in order to stabilize the body.

#### Comparison with other species

The morphology of MNs located in cervical, lumbar and sacral spinal segments have been investigated in several studies [45-48,84-87]. Comparison of these data showed differences between morphology and orientation of lumbosacral and cervical mammalian spinal MNs [46,84,88]. For example, the dendritic trees of hindlimb MNs presented more complex arborization pattern resulting in bigger length and surface area. The majority of cervical MNs had rostrocaudally oriented somata and dendritic bundle whereas the stellate-like dendritic trees of the lumbar MNs spread radially to almost all directions from the soma.

Comparison of our data on the morphology of lumbar MNs of frogs with MNs of cats showed several differences between the two species. The MNs in the frog presented smaller, elongated cell bodies emitting fewer stem dendrites. The size of the dendritic trees was about the half of that found in hindlimb innervating MNs of mammals [84,88]. Lumbar MNs in frogs, however, have more dendritic end points than similar MNs in the cat [42]. On the other hand, many more neurons control the functionally homologous muscles in cats than in frogs [89,90]. So, it appears that the higher complexity of individual MNs compensate for the smaller number of neurons and fewer individual dendrites/neuron in frog.

The organization of dendritic arbor seemed to be also different in lumbar MNs of frogs and cats. While the dendritic trees of cat MNs emerge in almost all directions without any obvious preference, the dendritic trees of frog MNs are organized into dorsal, lateral and dorsomedial groups. The lateral dendrites dendrites form a dense subpial meshwork running parallel with the border of the spinal cord. This subpial dendritic plexus is well developed in lower vertebrates including anurans but it is reduced in mammals.

### Electrical properties of limb moving motoneurons

Our major aim was to test if limb moving MNs are different electrically in the cervical and lumbar segments of the spinal cord. Factors shaping the electrical properties of neurons include the specific membrane resistance of the soma (R_ms_) and dedndrites (R_md_). However, the detailed membrane properties (R_ms_ and R_md_) of these large neurons are still not well known. In addition, the effective membrane resistance may be dependent on the activation state of synapses received by MNs in the functioning spinal networks. Therefore we used two approaches. First, we compared the METs of MNs by assigning different physiologically realistic somatic input resistances (1.4 or 5 MΩ) [16,18,19] with the assumption of homogeneous (R_ms_ = R_md_) and inhomogeneous (R_ms_ < R_md_) soma-dendritic membrane with a canonical R_md_ = 20000 Ωcm^2^ value for the dendrites (see Figures 6, 7, 8). In this part of the study our goal was to show that segmental differences exist between the METs of limb moving MNs independently of the detailed (and unknown) *inherent build up* of the soma-dendritic membrane (Figure 8) and the issue of synaptic background activity was not considered directly here. In this part we also illustrated the dependence of the rates of log attenuations (METs) on the possible inherent build ups (R_ms_-R_md_ pairs) of the same neuron (see Figure 6). In the second part of the study the *effects of synaptic background activity* (network activity) was investigated on the similarities/dissimilarities of voltage and current transfer properties of cervical and lumbar MNs by taking R_md_ = 20000 Ωcm^2^ as a control value. Higher and lower levels of synaptic activities were modelled by decreasing the R_md_ to 5000 Ωcm^2^ and increasing it to 50000 Ωcm^2^, while the R_ms_ was kept constant at 500 Ωcm^2^[29,33,34] (see Figures 9, 10, 11 and 12). This approach of modelling the intensity of general synaptic bombardment assumes that changes in network activity will primarily modify the activity of synapses received by dendrites and somatic membrane resistance remains largely unaffected. Consequently, the total neuron resistance (measured at the soma) is decreasing or increasing as synapses over the dendrites get more or less activated. This mechanism seems likely because of morphological and physiological reasons. Dendritic surface represents ~95–98% of the neuronal membrane and much more synapses are received by dendrites than by the soma in limb moving MNs of the frog [10]. Physiologically, Alaburda et al. [91] found phasic increases in conductance of MNs during scratch-like network activity in the isolated carapace-spinal cord preparation from turtles. Cortical neurons are also known to be in the “high-conductance state” in awake animals having lower neuron resistance than neurons recorded in slice preparations due to the constant synaptic bombardment in functioning cortical networks [92]. The functional (computational) consequences of the high-conductance states have been investigated extensively in neocortical neurons [92,93] but much less appreciated in the spinal cord.

#### Quantitative analysis of morphoelectrotonically transformed motoneurons

We started the investigation of electrotonic properties by performing morphoelectrotonic transformation (MET, [35]) on MNs to measure electrotonic distances between dendritic points and the soma. There are two advantages of this method over the classical way of measuring electrotonic distances. First, this measure of electrotonic distance takes into account that dendrites are not unbranched cylinders for which the standard definitions of space constant and electrotonic length were established many years ago [94], but a complex branching structure, which imposes unique boundary conditions for signal propagation in each dendritic branch. Secondly, the new definition of electrotonic distance in MET is additive, unlike the classical definition of electrotonic distance. This additive feature allows graphical display of dendritic arbors to visualize electrotonic distances in a more reliable manner. In the METs the distance between any dendritic point and the soma scales with the electrotonic distance of the two points, permitting a visual approach to passive signal transfer. This qualitative approach was first taken by Zador et al. [35] for different neurons of the brain and rarely followed by quantitative analysis of the METs (but see [76,95]). Here we went further and analyzed METs of MNs quantitatively for the first time and compared MNs responsible for moving the fore- and hindlimbs of frogs. The purpose was to find correlations between the morphological appearance and the METs of dendritic trees. We found significant differences in the combined dendritic lengths of dendritic trees of cervical and lumbar MNs in morphological sense, when distances were measured in μm, and also in the METs, when distances were measured in electrotonic lengths. These differences in METs of lumbar and cervical neurons were detected independently of the extent of inhomogeneity of the soma-dendritic membrane and the size of resistance of the MNs. However, the mean geometrical distances of branch points from the soma were the same in MNs of the two segments but they either differed or remained the same in the METs depending on the inhomogeneity of the soma-dendritic membrane and the neuron resistance.

Comparison of the METs with the original geometry of dendrites showed increasingly disproportionate changes in MET sizes in the more distant locations. This was observed independently of the size of soma-dendritic membrane inhomogeneity and the resistance of the neurons. According to the cable theory, in an infinite cylinder with passive membrane the voltage is decaying exponentially with distance if a steady current is injected (see [96] for a fuller discussion). Therefore, in this simple case, the relationship between the log attenuation (MET size) and the geometrical distance is linear. In our case, deviations from this linear relationship are due to the unique topology (see our results and [42]) of dendritic trees, which alter the boundary conditions for signal propagation. In addition to topology, dendritic diameters also play an important role in determination of the rates of attenuations from a given distance [29,97]. We showed that these morphological variables altered log attenuations differently in cervical and lumbar MNs, even if the same membrane properties were assumed, and alterations grew in size with the geometrical distance. These alterations from the original dendritic geometry were bigger in the lumbar MNs independently of the membrane properties. These results emphasize the complexity and location dependency of the relationship between geometry and dendritic signal propagation for limb moving MNs along the spinal cord.

Next, we investigated: 1) Which distance domains of the dendrites do differentiate between cervical and lumbar MNs and 2) How these distance domains do change when properties of the soma-dendrite membrane are altered?

Two distance domains, whose separation was at ~1500 μm distance, could be identified from where voltage attenuations to soma were characteristically related to the segmental origin of MNs. In this context we found the followings: i) PSPs propagating along the passive dendrites of cervical MNs attenuated less to soma than PSPs in lumbar MNs if the synapses were closer than ~1500 μm. The relationship is the opposite if PSPs were generated farther than ~1500 μm from the soma. ii) These findings are independent of the size of neuron resistivity and the inhomogeneity of the soma-dendritic membrane surface.

The observation that these relationships are independent of the size of neuron resistance suggests implications for the control of PSP attenuations by the uniform decrease or increase of background synaptic activity that changes the effective membrane resistance over the soma-dendritic membrane [29-31]. In this case, the homogeneous (or inhomogeneous; R_ms_ < R_md_) nature of the soma-dendritic membrane would be kept, while the neuron input resistance becomes lower with the overall increase in synaptic activity. However, the impact of background synaptic activity on membrane resistance is presumably bigger in dendrites than in the soma since ~95–98% of the membrane surface is given by the dendrites and the majority of synapses are received there in frog spinal MNs [10]. Consequently, the size of inhomogeneity in the soma-dendritic membrane may be decreasing with the increase in dendritic synaptic activity assuming an inherently lower resistance for the soma [20,26,27] and the neuron is becoming more like our homogeneous model. Changes in the size of inhomogeneity of the soma-dendritic membrane structure by the varying intensity of synaptic activity may then affect the rates of signal attenuations in the cervical and lumbar MNs but these changes will not modify whether dendrites of cervical or lumbar MNs are more effective in transfer of PSPs generated within the same distance domain. This suggests that neuron morphology may have selective control over the way how the rate of signal propagation changes with distance and with background synaptic activity in cervical and lumbar MNs in the frog.

#### Rates of voltage and current transfers differentiate limb moving motoneurons

Global comparison of log attenuations (MET) of cervical and lumbar MNs resulted in a nearly 100% segmentally correct classification by discriminant analysis. Electrical structures of MNs were further investigated by the voltage and current transfers in dendrites at different intensities of synaptic background activity. In these analyses we used standardized distributions of the transfer values to isolate structure-related electrical differences from those related purely to size. Such structural differences were suggested by the different numbers and distributions of branch points in MNs of the two spinal segments and by the different distance-dependence of log attenuations in the two groups of MNs.

This way, it was relevant to ask directly whether the electrotonic differences between fore- and hindlimb moving MNs are entirely due to their different metric (size-related) morphological properties or they are (also) consequences of the different dendritic structures.

Voltage transfer properties showed differences in the two groups of neurons both in steady-state and in case of transient signals. This suggests that identical inputs propagate differently in the dendrites of cervical and lumbar MNs and the level of depolarization reaching the soma or the nearby axon hillock may be different. Threshold potentials were investigated experimentally in putative MNs along the spinal cord in young frogs and no significant tendency of changes was detected according to the rostro-caudal positions of neurons [98] suggesting similar spiking thresholds in the cervical and lumbar spinal cord. Consequently, due to the different transfer properties of identical PSPs in MNs of the two spinal segments, the input–output properties will be different in the MNs innervating upper and lower extremities.

However, it is disputed if the major determinant of firing is to exceed a certain voltage threshold or to deliver enough current (charge) to the soma since experimental evidence exists to support each view depending on the conditions of action potential initiation (see [96] for a discussion). The amplitude of the somatic PSP is often small but the cell produces an action potential [28]. This led to the proposal that, under certain conditions, the time integral of the somatic EPSP might be a better single parameter that determines whether or not an action potential is generated [99,100]. This integral is proportional to the electrical charge reaching the soma. In this case the current transfer rather than the voltage transfer is the more relevant measure to relate input and output properties of the neuron. In our simulations steady-state current transfer segregated lumbar and cervical MNs under high synaptic activities.

The current transfer from a dendritic point to soma is equal to the rate of voltage transfer in the reverse (somatofugal) direction [35]. Our results on somatopetal current transfer properties are therefore directly related to the passive spread of back propagating potentials, a phenomenon has never been studied in vivo in frog spinal MNs but see [101] for rat spinal cord slice cultures.

Under steady-state conditions we detected a general tendency of increasing segregation (differences) between the limb moving MNs as the background synaptic activity was increased. This tendency was more pronounced if current rather than voltage transfers were considered. A similar tendency of increasing differences in electrotonic properties during higher synaptic activities was reported in a comparative study on different classes of spinal neurons, including MNs, in the cat [33].

Our findings on segmental segregation tendencies of limb moving MNs based on their morphological and dendritic signal transfer properties are strengthened by the consistency of the results obtained with the many different and validated statistical approaches, independently of the type of homogeneity index used, the choice of hierarchical cluster analysis and the weighting factors of descriptors.

## Conclusions

We showed location specificity of morphological and electrical transfer properties of the limb moving class of motoneurons in the frog spinal cord. Many of the location-specific differences were size-independent emphasizing the importance of *structural* differences in motoneurons along the rostro-caudal axis of the spinal cord. The location-dependent differences are likely to affect input–output properties of these MNs.

The results present the first detailed systematic analysis of the location-dependent properties of spinal motoneurons and suggest that specificity of locomotor networks, which control fore- and hind limb movements, is partly due to differences in their motoneurons.

These differences might reflect a basic initial segmental developmental pattern of MNs, which may then be refined according to the needs of specific muscles they innervate. This concept may obtain support from the experimental findings that the targets of these MNs are specified before the outgrowth of axon [52,102]. Further, variable expression of Hox genes along the rostro-caudal axis plays a role in the mechanism of target specification and segmental differentiation [51], which raises the possibility that these expression patterns may contribute to segmental specifications of morphological and electrical properties of MNs at this developmental stage [103-105].

## Abbreviations

ANOVA: Analysis of variance; BRP: Branch point of a dendrite; CNS: Central nervous system; ENDP: End point of a dendrite; EPSP: Excitatory postsynaptic potential; Hox: Homeobox; MET: Morphoelectrotonic transformation; MN: Motoneuron; PAST: Palaeontological statistics; PSP: Postsynaptic potential; SEM: Standard error of means; SPSS: Statistical package for the social sciences; λ: Space constant; g_max_: Peak synaptic conductance; t_max_: Time to peak of the synaptic conductance; R_md_: Specific dendritic membrane resistance; R_ms_: Specific somatic membrane resistance; R_N_: Neuron resistance.

## Competing interests

The authors declare that they have no competing interests.

## Authors’ contributions

AS wrote the program codes, run simulations, carried out statistical tests and contributed to drafting the paper. JS participated in running simulations and in data analysis. IW and AB reconstructed the neurons. AB also contributed to the body text. EW designed the study, participated in analysis and interpretation of data, and finalized the text. All authors read and approved the final manuscript.
